# Bioinformatic identification and characterization of human endothelial cell-restricted genes

**DOI:** 10.1186/1471-2164-11-342

**Published:** 2010-05-28

**Authors:** Manoj Bhasin, Lei Yuan, Derin B Keskin, Hasan H Otu, Towia A Libermann, Peter Oettgen

**Affiliations:** 1 Division of Interdisciplinary Medicine and Biotechnology, Beth Israel Deaconess Medical Center, Harvard Medical School, Boston MA 02215, USA; 2 Department of Medicine, Beth Israel Deaconess Medical Center, Harvard Medical School, Boston MA 02215, USA; 3 Division of Cardiology, Beth Israel Deaconess Medical Center, Harvard Medical School, Boston MA 02215, USA; 4 Division of Molecular and Vascular Medicine, Beth Israel Deaconess Medical Center, Harvard Medical School, Boston MA 02215, USA; 5 Department of Medical Oncology, Dana-Farber Cancer Institute, Boston, 02215 USA

## Abstract

**Background:**

In this study, we used a systematic bioinformatics analysis approach to elucidate genes that exhibit an endothelial cell (EC) restricted expression pattern, and began to define their regulation, tissue distribution, and potential biological role.

**Results:**

Using a high throughput microarray platform, a primary set of 1,191 transcripts that are enriched in different primary ECs compared to non-ECs was identified (LCB >3, FDR <2%). Further refinement of this initial subset of transcripts, using published data, yielded 152 transcripts (representing 109 genes) with different degrees of EC-specificity. Several interesting patterns emerged among these genes: some were expressed in all ECs and several were restricted to microvascular ECs. Pathway analysis and gene ontology demonstrated that several of the identified genes are known to be involved in vasculature development, angiogenesis, and endothelial function (P < 0.01). These genes are enriched in cardiovascular diseases, hemorrhage and ischemia gene sets (P < 0.001). Most of the identified genes are ubiquitously expressed in many different tissues. Analysis of the proximal promoter revealed the enrichment of conserved binding sites for 26 different transcription factors and analysis of the untranslated regions suggests that a subset of the EC-restricted genes are targets of 15 microRNAs. While many of the identified genes are known for their regulatory role in ECs, we have also identified several novel EC-restricted genes, the function of which have yet to be fully defined.

**Conclusion:**

The study provides an initial catalogue of EC-restricted genes most of which are ubiquitously expressed in different endothelial cells.

## Background

The endothelium, which lines the inner surface of all blood vessels, participates in several normal physiological functions including control of vasomotor tone, the maintenance of blood fluidity, regulation of permeability, formation of new blood vessels, and trafficking of cells [1]. The endothelium also plays an important role in several human diseases. In the setting of inflammation several genes become activated within the endothelium to facilitate the recruitment, attachment, and transmigration of inflammatory cells. Over time, however, in chronic inflammatory diseases EC responses become impaired, leading to EC dysfunction.

As a cell type, ECs exhibit tremendous heterogeneity [2]. For example, there are significant differences in EC structure and function based on the size and type of blood vessel, from larger arteries or veins, to medium sized arterioles or venules, down to capillary ECs. There is also significant heterogeneity at the level of a particular tissue or organ. For example, in the brain, the endothelium plays a particularly important protective role as part of the blood brain barrier with ECs that are closely attached to one another forming a tight barrier that is impermeable to the passage of even small solutes or ions. In contrast, in the liver, the sinusoidal ECs are fenestrated so that small to moderate size transcellular pores promote the uptake of large lipid containing particles from the blood [3,4].

The endothelium is known to play an important role in several human diseases including atherosclerosis, diabetes mellitus, and sepsis. The overall goal of the current study was to use primary and publicly available microarray data from human ECs, non ECs, and tissues, to identify genes that exhibit an EC-restricted pattern, define their distribution in different tissues, and determine whether changes in the expression of any of the genes are linked to particular diseases. Our study, has for the first time, identified and ranked a significant number of genes that exhibit an EC-restricted expression pattern. Among these genes, several interesting patterns of expression emerge. Whereas many of the genes are expressed in all ECs, some are restricted to microvascular ECs. The vast majority of EC-restricted genes are expressed in multiple tissues. The EC-restricted genes were found to be associated with a number of different cellular functions including vasculature development, cell differentiation, and angiogenesis. Analysis of the regulatory regions of the EC-restricted genes demonstrated enrichment of binding sites for a selected number of transcription factors and microRNAs.

## Methods

### Cell culture

HUVEC (human umbilical vein EC cell; Lonza), HAEC (human aortic EC cells), HCAEC (human coronary artery EC cells), HPAEC (human pulmonary artery EC cells), and HMVEC (human microvascular (dermal) EC cell; kindly provided by Dr. William Aird) were grown in EBM-2 (EC Cell Basal Medium-2) supplemented with EGM SingleQuots (Lonza). HASMC (human aortic smooth muscle cell) were grown in SmBM Basal Medium supplemented with SmGM-2 SingleQuot (Lonza). For the isolation of the T and B cells, discarded leukocytes from platelet donations by healthy human donors were used in this study. Samples were obtained from subjects after informed consent was obtained using an institutionally approved protocol (IRB protocol 2005-P-001364/2). Red blood cells were removed using Ficoll-Paque PLUS according to manufacturer's protocol. (GE-Healthcare. Uppsala Sweden). Donor Peripheral Blood Mononuclear Cells (PBMC) were stained with pan T-cell specific CD3-PE and pan B-cell specific CD20-FITC antibodies. Fluorescently labeled cells were sorted using a high speed cell sorter. (FACS Aria. BD biosciences San Jose. California).

### RNA isolation

Total RNA was isolated using the RNAeasy kit (QIAGEN) following the manufacturer's instructions.

### Microarray Analysis

Transcriptional profiling of endothelial and non-EC cells was performed using the Affymetrix oligonucleotide microarray HT U133 plate with 24 chips according to a standard Affymetrix protocol for cDNA synthesis, *in vitro *transcription, production of biotin-labeled cRNA, hybridization of cRNA with HT Plate A and B, and scanning of image output files [5]. The quality of hybridized chips was assessed using Affymetrix guidelines on the basis of average background, scaling factor, number of genes called present, 3' to 5' ratios for beta-actin and GAPDH and values for spike-in control transcripts [6]. We also checked for reproducibility of the samples by using chip to chip correlation and signal-to-noise ratio (SNR) methods for replicate arrays. All the high quality arrays were included for low and high level bioinformatics analysis. Primary gene expression data are publicly available at GEO http://www.ncbi.nlm.nih.gov/geo/ in GSE21212.

### Statistical Analysis

To obtain the signal values, high quality chips were further analyzed by dChip, as it is more robust than MAS5.0 and RMA in signal calculation. The raw probe level data was normalized using smoothing-spline invariant set method. The signal value for each transcript was summarized using PM-only based signal modeling algorithm described in dChip. The PM only based modeling based algorithm yields less number of false positives as compared to the PM-MM model. In this way, the signal value corresponds to the absolute level of expression of a transcript[7]. These normalized and modeled signal values for each transcript were used for further high level bioinformatics analysis. During the calculation of model based expression signal values, array and probe outliers are interrogated and image spikes are treated as signal outliers.

When comparing two groups of samples to identify genes enriched in a given phenotype, if the 90% lower confidence bound (LCB) of the fold change (FC) between the two groups was above 3 and median false discovery rate is <2%, the corresponding gene was considered to be differentially expressed [8]. LCB is a stringent estimate of FC and has been shown to be the better ranking statistic [9]. It has been suggested that a criterion of selecting genes that have an LCB above 2.0 most likely corresponds to genes with an "actual" fold change of at least 3 in gene expression [8,10].

### Identification of EC-restricted genes

The list of differentially expressed genes obtained from the primary analysis (previous section) was further analyzed through a series of steps to obtain EC-restricted genes. This analysis was performed using the following three steps, i); determination of the enrichment score, ii); performing an outlier analysis, and iii); ranking the genes according to EC specificity.

### i) Enrichment Score [ECS]

The enrichment analysis was performed to determine the probability that genes are specifically over expressed in ECs as compared to other primary non-ECs. For this analysis we used the public REFEXA database http://www.lsbm.org/site_e/database/index.html. The REFEXA database consists of gene expression data from a series of primary cells, cancer cell lines, and tissues. The MAS5 normalized data was manually obtained from the database for all the transcripts that were identified as highly expressed in ECs compared to non-ECs in the primary analysis. The enrichment score of each gene was determined by calculating the relative expression in the ECs compared to non-ECs. Each transcript was assigned a present/absent call in every primary cell on the basis of expression value. The transcript is called present (P) in a primary non-endothelial cell if it was expressed >50% of the expression level in the primary ECs, otherwise it was called absent (A). The EC score (ECS) is obtained using the following equation:(1)

where ECS_j _is the enrichment score for a transcript j, A_i _and P_i _are the present and absent calls for the transcript in different normal primary cells (n).

### ii) Outlier Analysis

The outlier analysis was performed on the list of genes obtained after step i) for the selection of genes with restricted EC expression. The outlier analysis was performed by means and standard deviation of the expression values using publicly available microarray data. If the expression of a given transcript in a sample falls 2 standard deviations outside of the mean expression in the distribution obtained using all samples, the particular sample is considered as an outlier. If the cluster of the outliers consists only of ECs, the genes were considered as good candidates for being EC-restricted. On the contrary, if the cluster of the outliers consists of ECs and non-ECs, these genes were considered to have less specificity for ECs and were filtered out from the final analysis.

### iii) Ranking of EC-restricted genes

After the outlier and enrichment analysis, all the identified EC-restricted genes were ranked on the basis of average fold change of a gene in ECs as compared to non ECs (REF_FOLD) in publicly available datasets (REFEXA) and Fold change between ECs and non-ECs from our primary experiment (FC) [EQ 2]. The genes with high REF_FOLD and high FC are considered to be more EC-restricted and assigned a higher rank.(2)

where REF_FOLD = (Expression in ECs in public set/Expression in Non-EC) and FC = (Expression in ECs in primary set/Expression in Non-EC).

To further reduce the false positive rate, we have selected the top 60% of the transcripts with greater than 3 fold expression in ECs compared to non-ECs as good candidates for endothelial restriction.

### Pathways, Gene ontology and Disease set enrichment analysis of EC-restricted genes

The functional analysis of the EC-restricted genes was performed in terms of canonical pathways, disease sets and gene ontology (GO) categories. The canonical pathways and disease set enrichment analysis was performed using the MetaCore tool of GeneGo package http://www.genego.com/. It consists of manually curated information about gene regulation, protein interactions, and metabolic and signaling pathways. The overrepresented canonical pathways and disease biomarker sets were ranked on the basis of P values obtained using the Simes procedure accounting for multiple hypothesis testing representing the probability of mapping arising by chance, based on the number of EC-restricted genes identified in a particular canonical pathway or disease compared to the total number of genes in the GO category/Disease set. The Go categories/Disease set with a False Discovery Rate (FDR) corrected P value <0.05 were considered significant.

The Database for Annotation, Visualization and Integrated Discovery (DAVID) was used to identify over-represented gene ontology categories form the endothelial restricted genes [11]. DAVID is an online implementation of the EASE software that produces the list of overrepresented categories using jackknife iterative resampling of the Fisher exact probabilities. A score was assigned to each category by using "-log" of EASE score to show the significantly enriched gene ontology categories. The related gene ontology categories were merged into a cluster using the functional clustering module of DAVID. Higher enrichment scores for particular genes reflect increasing confidence in their over-representation.

### Analysis of transcription factor binding sites

Recent improvements in bioinformatics methods for the analysis of sequences regulating transcription have made it possible to elucidate potential factors involved in regulating key regulatory networks underlying a transcriptional response. We divided the EC specific genes into two sets on the basis of K Mean clustering for promoter analysis i) high expression in all ECs ii) and high expression in HMVEC. The promoter analysis was performed separately on these two sets using the online tool ExPlain http://explain.biobase-international.com/cgi-bin/biobase/ExPlain_2.4.2/ for detection of over-represented transcription factor binding sites. ExPlain uses the MatchTM, a weight matrix-based tool for searching putative transcription factor binding sites [12,13].

For the analysis, we selected regions from 2000 bp upstream to 100 bp downstream of the transcription start site of each gene (Yes set). The enrichment was obtained against a random set of promoters obtained from human housekeeping genes (No set). The entire vertebrate non-redundant set of transcription factors matrix from transfac database was used for scanning potential binding sites [14]. The matrices that did not differ much in density between the positive and negative set were removed from the results. A significant over-representation of a transcription factor binding site in a target set as compared to the background set was determined using a 1-tailed Fisher exact probability test *[P value < 0.01,FC (yes_set/no_set) > 1.2)*. After completion of the enrichment analysis, the transcription factor binding sites for each set were compared with each other, in order to identify TF binding sites that were common and distinct among the different types of ECs (e.g. all, microvascular).

### MicroRNA target analysis

Another potential mechanism of regulating EC specific genes could be through miRNA, a class of small non-coding RNAs, that regulate gene expression primarily through post-transcriptional repression by promoting mRNA degradation in a sequence-specific manner [15]. We were interested in identifying whether miRNA binding sites are enriched in EC-restricted genes. Computational analysis of the miRNA targets sites was performed using **C**omposite **R**egulatory **S**ignature **D**atabase (**CRSD**) http://140.120.213.10:8080/crsd/main/home.jsp, a comprehensive server for composite regulatory signature discovery. CRSD has a package for prediction of miRNA binding sites by searching the UTRs for segments of perfect Watson-Crick in the 3'UTR of the target gene set [16].The miRNA binding sites for each of the micro RNA are calculated in the EC-restricted set and the background set (54,576 genes from human unigenes). The enrichment of each miRNA binding site is calculated on the basis of its abundance in the EC-restricted set and the background set. The significance of enrichment is expressed as a P value (smaller the P value more significant is the enrichment).

### Tissue specificity of EC specific Gene

In order to determine the normal tissue distribution of the EC specific genes, we obtained the normalized expression level from the Stanford Source database [17]. Source database presents the relative expression level of a gene in different tissues that is normalized for the number of samples from each tissue included in UniGene. The gene expression information for the different transcripts was obtained from dbEST expression profile.

In addition to relative gene expression information from the Source database, we have also manually curated the protein expression information about the endothelial specific genes from the Human Protein Atlas database. The Human Protein Atlas is a comprehensive database that provides the protein expression profiles for a large number of human proteins, presented as immunohistological images from most human tissues [18,19]. It contains antibody-based protein expression and localization profiles of >4,000 proteins in 48 normal human tissues and 20 different cancers [20]. The expression level of each protein is presented in a four color scale system that takes into consideration the intensity of the protein expression and quantity of positive images tested for each protein. It is a very useful tool to extract the relative expression level of proteins in different tissues.

### Quantitative real-time PCR

Total RNA was isolated using the RNAeasy kit (QIAGEN, Valencia, CA). Single stranded cDNA was synthesized from total RNA using High Capacity RNA-to-DNA Kit (Applied Biosystems). SYBR Green I-based real-time PCR was carried out on an Opticon Monitor. The sequences of the primers used in this study are listed in Additional File 1. For normalization of each sample, human specific TATA-binding protein (TBP) primers were used to measure the amount of TBP cDNA.

## Results

### Identification of EC-restricted genes

**Figure 1:**
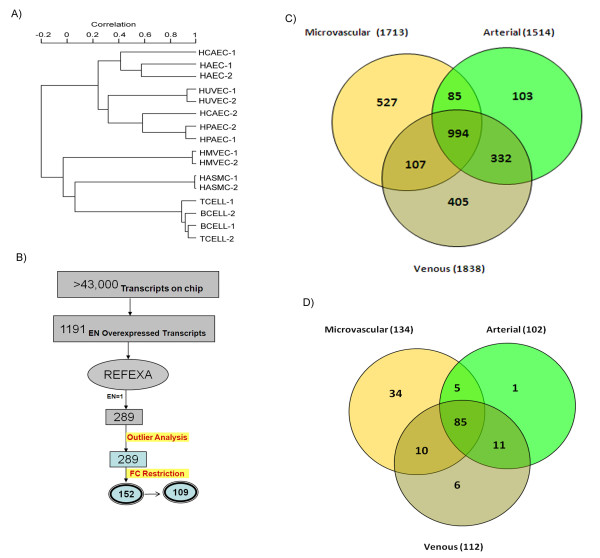
**Overall approach for extraction of endothelial restricted genes**. A) Unsupervised Pearson Correlation based cluster of different EC and non-EC arrays after normalizing the data. ECs (HMVEC, HUVEC, HPVEC, HAEC, and HCEAC) form separate clusters from non-ECs (HSAMC, B Cells, T Cells). In most of cases biological replicates of each cell type have better correlation with each other than with other cell types. B) Venn diagram indicating overlap between microvascular, arterial and venous endothelial differentially expressed genes obtained from the primary analysis C) Schematic representation of the approach for identifying the genes with EC-restricted expression (EC-restricted) D) Venn diagram depicting the overlap between microvascular, arterial and venous endothelial restricted transcripts.

In an effort to identify genes that exhibit an EC-restricted pattern total RNA was isolated from primary cultured ECs (including HUVEC, HPAEC, HAEC, HMVEC, and HCAEC) and non-ECs (HASMC, B cells, T cells). Gene expression profiling was performed using a high throughput platform, HT U133 plate, that measures more than 43,000 well-characterized genes and UniGene clusters. The expression profiling was performed in duplicate. All the array data was determined to be of high quality as assessed by the scaling factor, average background, percent present calls, and 3'/5'RNA ratio. After normalization and preprocessing of the data, we generated a list of genes that are significantly differentially expressed between different ECs and non-ECs. The heterogeneity in the transcription profile of the EC was identified using unsupervised clustering, reflecting the global similarities between the samples [Figure 1A]. Unsupervised clustering demonstrated the highest similarity within the biological replicates and the least similarity between ECs and non-ECs. The cladogram produced by unsupervised clustering depicted that venous and pulmonary arterial ECs are much closer in expression profile as compared to microvascular cells.

Comparing groups, we found 1,713 transcripts that are differentially expressed in HMVEC compared to non-ECs (LCB > 3 and FDR < 2%). Similarly for HUVEC and HPVEC, 1,534 and 1,539 transcripts were respectively differentially expressed compared to non-ECs. For the arterial EC cells, 1,239 HCAEC and 1,316 HAEC transcripts were determined to be differentially expressed in these cells compared to non-ECs. Comparison of the differentially expressed transcripts in microvascular (HMVEC), venous (HUVEC, HPVEC) and arterial (HAEC, and HCAEC) cells using Venn diagrams revealed that approximately half of the transcripts are differentially expressed in all three EC types. However we also observed that each EC type possessed a unique expression signature; the differential expression of transcripts was limited to one type of EC [Figure 1B].

**Figure 2:**
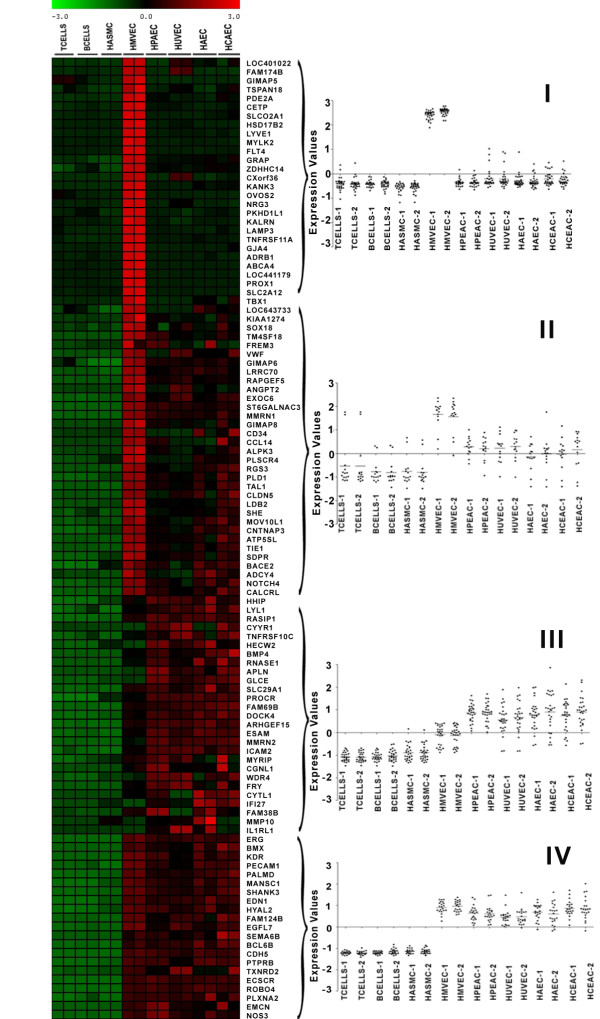
**Colorgram depicting the expression of EC-restricted genes in different cell types in the primary set**. The columns represent the samples and rows represent the genes. Gene expression is shown with a pseudocolor scale (-3 to 3) with red color denoting high expression level and green color denoting low expression level of the gene. The scatter plots along the heatmap depict the different patterns in expression of EC-restricted genes obtained using K mean clustering. The K mean clusters are represented as scatter plots with bars denoting the mean expression level. Pattern I and IV depict a range of expression patterns exhibited by EC-restricted genes. For example pattern IV and I denotes the genes that are highly expressed in all endothelial cell types (pan EC) and HMVEC cells respectively.

**Figure 3:**
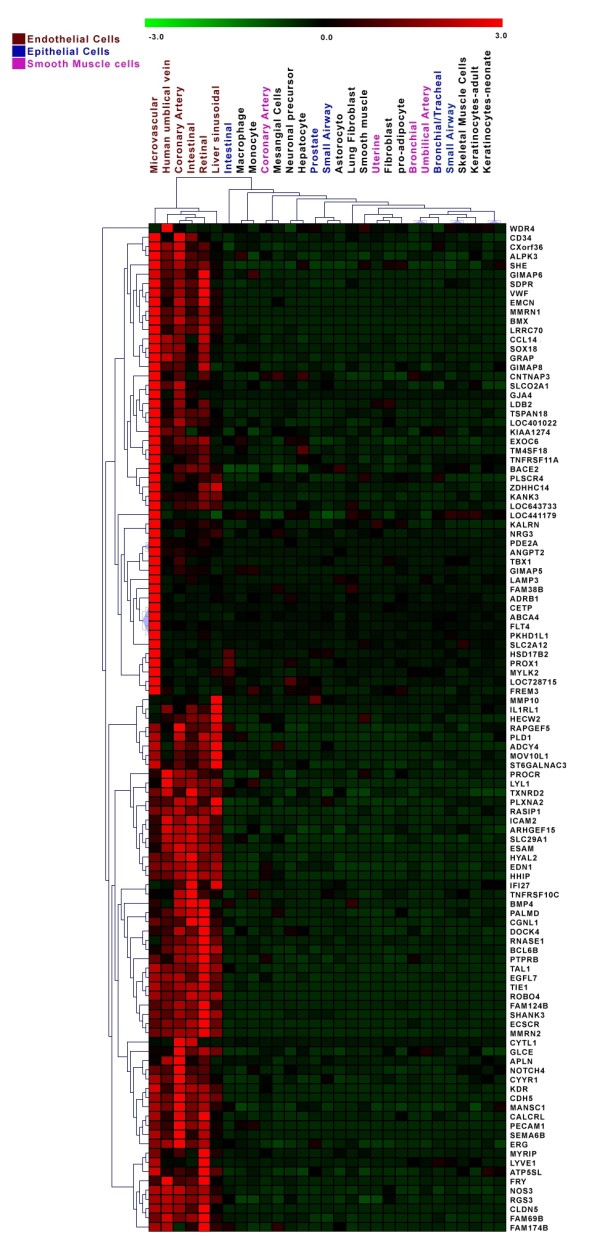
**Expression of ECs restricted genes in REFEXA database**. **A) **Hierarchical clustering analysis of EC-restricted expression genes using REFEXA gene expression data. The columns represent the samples (primary endothelial and non endothelial cells from REFEXA database) and rows represent the genes. The detailed information about the primary cells can be obtained from REFEXA database http://157.82.78.238/refexa/main_search.jsp. Gene expression is shown with pseudocolor scale (-3 to 3) with red denoting high expression level and green denoting low expression level of gene.

**Figure 4:**
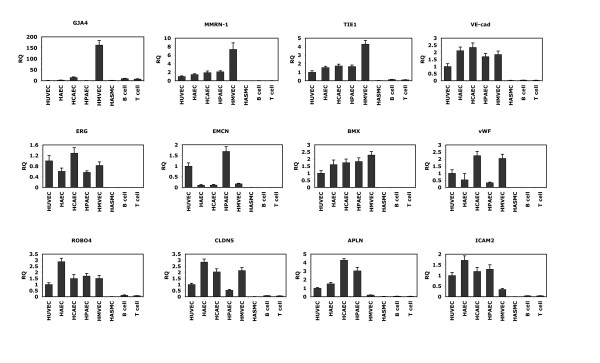
**Validation of a selected subset of endothelial-restricted genes by quantitative RT-PCR**. Validation of a subset of EC-restricted genes from Table 1 was conducted using primary ECs and non-ECs by quantitative RT-PCR (n = 3 per cell type). The gene symbol is listed for each gene. RQ refers to "relative quantity" where the expression in HUVECs has been set to 1.0 and the relative expression of the other cell types are compared to that in HUVECs.

The total number of transcripts that are significantly different in at least one of the EC types compared to non-ECs consists of 2553, representing 1617 genes. To further refine our initial list of EC-restricted genes, we evaluated the expression of these genes using the data from REFEXA http://www.lsbm.org/site_e/database/index.html to identify EC-restricted genes. To calculate an enrichment score for each gene, expression values were manually obtained for each transcript using the REFEXA database http://www.lsbm.org/site_e/database/index.html. This database has MAS5 normalized gene expression data for several primary cells, including ECs, cancer cell lines, and normal tissues. For analysis we only used the expression data for 30 primary cells and excluded all cancer cell lines. The enrichment and outlier analysis identified 289 outlier transcripts with an enrichment score of 1 (see methods for details). To further reduce the number of false positive results, the top 60% (168 transcripts) of transcripts with an average of greater than or equal to 3 fold overexpression in EC cells as compared to non-EC cells were considered EC-restricted. The expression value of these 168 transcripts was manually checked and transcripts with reduced specificity were removed. After manual inspection of relative expression profiles of each transcript, we selected 152 transcripts that correspond to 109 valid genes exhibiting an EC-restricted pattern (Table 1). The 152 transcripts with varying EC specificity are ranked on the basis of fold change in the primary set and fold change from the external datasets (e.g. REFEXA). The Rank score is a significance level with larger rank scores indicating increasing confidence in endothelial restriction. The overall schema of curating endothelial specific genes is shown in Figure 1C. Many genes that are known to be EC-restricted, including angiopoietin-2, von Willebrand's factor (vWF), VE-cadherin (CD144) are at the top of the list (Table 1). Comparison of the EC-restricted transcripts in microvascular (HMVEC), venous (HUVEC, HPVEC) and arterial (HAEC, and HCAEC) cells using Venn diagrams revealed that most of the transcripts are differentially expressed in all three EC cell types. Only a small fraction of transcripts are uniquely differentially expressed in microvascular ECs [Figure 1D]. A colorogram demonstrating the expression pattern for each of the EC-restricted genes is shown in Figure 2. The colorogram consists of a range of patterns from transcripts highly expressed in all EC types (Pattern IV) to transcripts that are highly expressed in particular EC types (Pattern I). ANGPT2, TBX1, FLT4 are examples of genes that are highly expressed in the HMVEC cells. The expression patterns of EC-restricted genes were further confirmed using the REFEXA dataset [Figure 3]. To further validate the microarrays results, we used PCR to quantitate the expression levels of 12 randomly selected EC-restricted genes in primary ECs and non-ECs. A very similar EC-restricted expression pattern was observed for all 12 genes [Figure 4]. Although the relative fold enrichment of some of the EC-restricted genes was somewhat lower than initially identified by microarray analysis, the expression in non-ECs remained quite low or absent in comparison to ECs.

**Table 1:** List of the endothelial restricted genes with detailed annotation and rank score

Probe	Gene Symbol	UGCluster	FC	REFEXA_FC	Rank
205612_at	MMRN1	Hs.268107	172.65	64.625	11157.50625
204482_at	CLDN5	Hs.505337	124.75	47.5625	5933.421875
202112_at	VWF	Hs.440848	61.15	78.05172414	4772.862931
206464_at	BMX	Hs.495731	55.28	80.46153846	4447.913846
205572_at	ANGPT2	Hs.583870	69.88	20.21875	1412.88625
204904_at	GJA4	Hs.296310	37.23	34.5	1284.435
204677_at	CDH5	Hs.76206	79.89	13.96610169	1115.751864
204468_s_at	TIE1	Hs.78824	78	14.19626168	1107.308411
226028_at	ROBO4	Hs.524121	34.97	27.89130435	975.358913
227779_at	ECSCR	Hs.483538	53.73	12.03448276	646.6127586
222856_at	APLN	Hs.303084	76.9	8.106666667	623.4026667
207526_s_at	IL1RL1	Hs.66	64.53	9.56846473	617.453029
204134_at	PDE2A	Hs.503163	43.51	13.72368421	597.1175
214319_at	FRY	Hs.507669	38.96	15.2962963	595.9437037
219059_s_at	LYVE1	Hs.655332	88.96	6.384615385	567.9753846
201785_at	RNASE1	Hs.78224	28.82	18.38461538	529.8446154
236262_at	MMRN2	Hs.524479	28.44	18.28571429	520.0457143
204818_at	HSD17B2	Hs.162795	133.64	3.846938776	514.104898
220637_at	FAM124B	Hs.147585	17.93	23.09090909	414.02
229902_at	FLT4	Hs.646917	19.45	20.94594595	407.3986486
205392_s_at	CCL14	Hs.714858	45.05	8.48	382.024
223567_at	SEMA6B	Hs.465642	17.28	21.82608696	377.1547826
211273_s_at	TBX1	Hs.173984	35.43	10.18181818	360.7418182
213715_s_at	KANK3	Hs.322473	34.78	10.23076923	355.8261538
222885_at	EMCN	Hs.152913	36.24	8.930555556	323.6433333
206331_at	CALCRL	Hs.470882	44.19	7	309.33
225369_at	ESAM	Hs.173840	14.83	20.10714286	298.1889286
219837_s_at	CYTL1	Hs.13872	47.07	5.946969697	279.9238636
241926_s_at	ERG	Hs.473819	79.8	3.44	274.512
238488_at	LRRC70	Hs.482269	12.1	21	254.1
203934_at	KDR	Hs.479756	40.75	6.224137931	253.6336207
227923_at	SHANK3	Hs.149035	15.6	12.96296296	202.2222222
225817_at	CGNL1	Hs.148989	27.25	7.260869565	197.8586957
229002_at	FAM69B	Hs.495480	26.39	7.414634146	195.6721951
228489_at	TM4SF18	Hs.22026	53.73	3.328	178.81344
210082_at	ABCA4	Hs.416707	16.65	9.857142857	164.1214286
235334_at	ST6GALNAC3	Hs.337040	24.36	6.659090909	162.2154545
229309_at	ADRB1	Hs.99913	19.91	7.891891892	157.1275676
235044_at	CYYR1	Hs.37445	23.53	5.6	131.768
205569_at	LAMP3	Hs.518448	14.72	8.56	126.0032
229233_at	NRG3	Hs.125119	19.5	6.285714286	122.5714286
235050_at	SLC2A12	Hs.486508	17.4	6.363636364	110.7272727
220027_s_at	RASIP1	Hs.233955	16.99	6.03125	102.4709375
204683_at	ICAM2	Hs.431460	9.09	11.23245614	102.1030263
220300_at	RGS3	Hs.494875	29.92	3.411764706	102.08
206283_s_at	TAL1	Hs.705618	24.65	4.097222222	100.9965278
227307_at	TSPAN18	Hs.385634	7.32	13.53333333	99.064
206210_s_at	CETP	Hs.89538	7.91	12.33333333	97.55666667
228601_at	LOC401022	Hs.98661	12.94	7.36	95.2384
218825_at	EGFL7	Hs.91481	13.85	6.789473684	94.03421053
211518_s_at	BMP4	Hs.68879	28.88	3.243243243	93.66486486
229726_at	GRAP	Hs.567416	9.1	10.14285714	92.3
229376_at	PROX1	Hs.585369	26.82	3.338461538	89.53753846
204368_at	SLCO2A1	Hs.518270	17.02	5.051724138	85.98034483
230132_at	ATP5SL	Hs.351099	22.49	3.585106383	80.62904255
209543_s_at	CD34	Hs.374990	17.19	4.68115942	80.46913043
228311_at	BCL6B	Hs.22575	12.6	6.333333333	79.8
219568_x_at	SOX18	Hs.8619	3.72	21.42857143	79.71428571
218736_s_at	PALMD	Hs.483993	17.5	4.270072993	74.72627737
204681_s_at	RAPGEF5	Hs.174768	22.02	3.351851852	73.80777778
239665_at	LOC441179	Hs.719702	20.51	3.578947368	73.40421053
238846_at	TNFRSF11A	Hs.204044	11.24	6.416666667	72.12333333
222911_s_at	CXorf36	Hs.98321	18.06	3.953703704	71.40388889
231887_s_at	KIAA1274	Hs.202351	13.86	4.685185185	64.93666667
202411_at	IFI27	Hs.532634	8.4	7.202764977	60.50322581
205581_s_at	NOS3	Hs.647092	11.89	4.958333333	58.95458333
206481_s_at	LDB2	Hs.714330	18.81	3.117505995	58.64028777
224385_s_at	MOV10L1	Hs.62880	5.37	10.09090909	54.18818182
230250_at	PTPRB	Hs.434375	13.61	3.787878788	51.5530303
230673_at	PKHD1L1	Hs.170128	4.04	12.625	51.005
222908_at	FAM38B	Hs.585839	7.79	6.5	50.635
240646_at	GIMAP8	Hs.647121	8.59	5.7	48.963
231792_at	MYLK2	Hs.86092	13.89	3.5	48.615
208983_s_at	PECAM1	Hs.514412	9.63	4.606138107	44.35710997
51158_at	FAM174B	Hs.27373	12.38	3.533333333	43.74266667
214156_at	MYRIP	Hs.594535	3.08	12.88888889	39.69777778
205507_at	ARHGEF15	Hs.443109	11.7	3.202898551	37.47391304
218901_at	PLSCR4	Hs.477869	7.29	5.044217687	36.77234694
228342_s_at	ALPK3	Hs.459183	12.23	3	36.69
219247_s_at	ZDHHC14	Hs.187459	4.32	8.176470588	35.32235294
213552_at	GLCE	Hs.183006	10.2	3.446428571	35.15357143
205247_at	NOTCH4	Hs.436100	6.44	5.409090909	34.83454545
205003_at	DOCK4	Hs.654652	11.06	3.085365854	34.12414634
218711_s_at	SDPR	Hs.26530	8.83	3.822222222	33.75022222
201801_s_at	SLC29A1	Hs.25450	10.62	3.057324841	32.46878981
218995_s_at	EDN1	Hs.511899	9.37	3.31779661	31.08775424
206855_s_at	HYAL2	Hs.76873	5.2	5.645502646	29.35661376
226636_at	PLD1	Hs.382865	8.18	3.347826087	27.38521739
211177_s_at	TXNRD2	Hs.443430	6.85	3.825688073	26.2059633
228245_s_at	OVOS	Hs.524331	6.8	3.851485149	26.19009901
218805_at	GIMAP5	Hs.647079	4.08	6.153846154	25.10769231
233924_s_at	EXOC6	Hs.655657	6.79	3.680672269	24.99176471
223796_at	CNTNAP3	Hs.658328	7.27	3.396039604	24.68920792
220945_x_at	MANSC1	Hs.591145	8.04	3.065934066	24.65010989
230800_at	ADCY4	Hs.443428	7.13	3.260869565	23.25
237466_s_at	HHIP	Hs.507991	5.84	3.927492447	22.93655589
205635_at	KALRN	Hs.8004	4.8	4.657894737	22.35789474
240890_at	LOC643733	Hs.713751	3.71	5.5	20.405
213030_s_at	PLXNA2	Hs.497626	5.18	3.504761905	18.15466667
243337_at	FREM3	Hs.252714	3.42	4.461538462	15.25846154
226882_x_at	WDR4	Hs.248815	3.51	4.122807018	14.47105263
232080_at	HECW2	Hs.654742	4.02	3.578947368	14.38736842
210044_s_at	LYL1	Hs.46446	4.42	3.239669421	14.31933884
205680_at	MMP10	Hs.2258	4.03	3.326693227	13.40657371
206222_at	TNFRSF10C	Hs.655801	3.29	3.714285714	12.22
219777_at	GIMAP6	Hs.647105	3.64	3.335664336	12.14181818
203650_at	PROCR	Hs.647450	3.27	3.582278481	11.71405063
222446_s_at	BACE2	Hs.529408	3.26	3.023094688	9.855288684
238036_at	SHE	Hs.591481	3.06	3.219512195	9.851707317

### Pathways and Gene Ontology (GO) Processes modulated by EC-restricted genes

**Figure 5:**
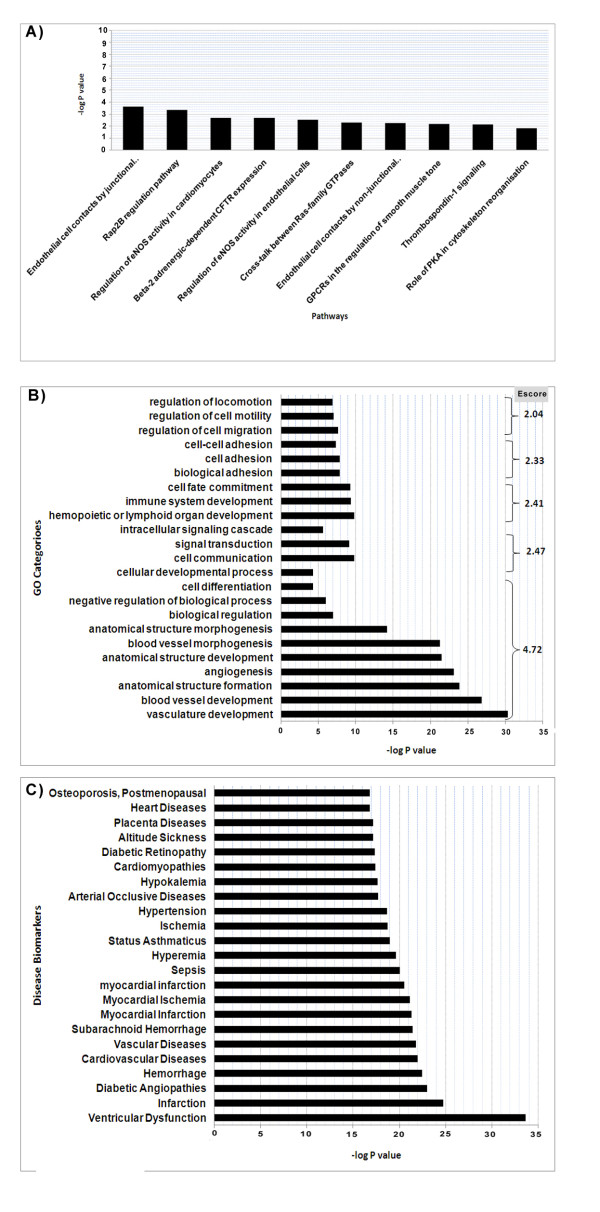
**Enrichment analysis of EC-restricted genes**. **A) **Top enriched Canonical Pathways **B) **Top enriched GO Processes. **C) **Top enriched disease set. The analysis for pathways and disease set enrichment was performed using the MetaCore tool of the GeneGo package. The GO categories enrichment analysis was performed using the DAVID tool. The Bar graphs depict the enriched pathway or Go process categories and -log of the P value. The P value depicts the significance of enrichment, the smaller is the P value the more significant is the enrichment. The pathways and disease sets with FDR adjusted P value < 0.05 are considered significant. The panel for gene ontology enrichment depicts the enrichments for each GO category (-log P value) as well as the Escore for a cluster of related GO categories.

We performed an enrichment analysis of the EC-restricted genes to identify the pathways and GO processes where the EC-restricted genes occur more often than would be expected by random distribution. The pathway enrichment analysis was performed using the MetaCore tool of the GeneGO package where P values of <0.05 (FDR adjusted) are considered significant. The enrichment analysis identified a set of statistically significant enriched pathways (Figure 5A). The most highly enriched pathways included "*EC contacts by junctional/nonjuctional mechanisms*", "*Regulation of eNOS activity in cardiomyocytes and endothelial cells*", "*thrombospondin signaling*", "*Role of PKA in cytoskeleton reorganization*", many of which would be expected based on the identified gene list. The enrichment analysis for GO categories was performed using the Database for Annotation, Visualization and Integrated Discovery (DAVID) program. The top clusters of biological processes and metabolic functions that are enriched in the set of differentially expressed genes are shown in Figure 5B. The most highly enriched clusters of the gene ontology categories included vasculature development and angiogenesis, immune responses, cell adhesion, and cell motility and migration. Vascular development and angiogenesis is the highest enriched GO cluster in which the EC-restricted genes are overrepresented (Enrichment score 4.72). This finding supports the overall concept that at least a subset of the genes we identified as being EC-restricted have previously been described in processes known to involve ECs.

#### Disease set enrichment of EC-restricted genes

In order to evaluate whether the EC-restricted genes are potentially linked to the pathogenesis of certain human diseases, we performed a disease set enrichment analysis using disease sets on the basis of published literature (DSPL). DSPL enrichment analysis was performed using the MetaCore tool in the GeneGO package. The disease associations are summarized in Figure 5C, depicting the top diseases in which EC-restricted are enriched. The EC-restricted genes are enriched in the many cardiovascular diseases including ventricular dysfunction, myocardial infarction, hypertension, diabetic angiopathies, arteriosclerosis, and several other vascular diseases. Interestingly, ischemia was listed as a disease in which the EC-restricted are over-represented (P value = 2E-06). The EC-restricted genes are also enriched (P value < 0.01) in neurological diseases including subarachnoid hemorrhage (P value = 3.00E-07).

### Regulatory mechanism governing EC-restricted genes

**Figure 6:**
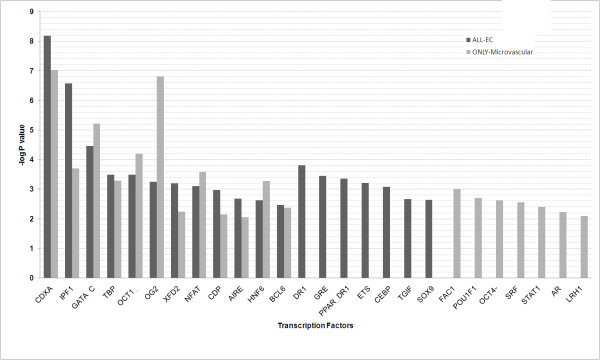
**Regulation analysis of EC-restricted genes**. The list of the transcription factor binding sites that are enriched in 2 kb upstream to 100 bp downstream region. The enrichment in gene sets that are highly expressed in all endothelial cells and only microvascular EC is shown in black and grey color respectively. The X-axis represents the transcription factors and Y-axis represents -log P value.

To begin to understand the complex and intricate regulation of the EC-restricted genes, we were interested in determining whether certain transcription factors or miRNAs might be involved in regulating these genes. Transcription factors play a critical role in defining cell and tissue specificity of gene expression. In this study the TFactor enrichment analysis was performed on two sets of EC-restricted genes categorized on the basis of expression profiles; the sets of genes are highly expressed in i); all EC types (pan EC), ii); only in HMVEC. The TFactor enrichment analysis was only performed on these two sets as they constitute the major fraction of EC-restricted genes. TFactor enrichment analysis was performed using the ExPlain tool, a program for gene expression analysis from BIOBASE. We performed the analysis on a region 2 kb upstream to 100 bp downstream of each of the EC-restricted genes using vertebrate_non_redundant matrices (yes set). Background frequencies were calculated based on the promoters of human housekeeping genes (No set) [12]. A TF binding site was considered to be enriched in a gene set on the basis of the P value (P value < 0.001 and Yes/No > 1.2). The analysis identified binding sites for >20 transcription factors, among the EC-restricted genes expressed in all EC, and in the subset enriched only in microvascular ECs [Figure 6]. Binding sites for the TF factor that were identified for both of these sets of genes included, CDXA, GATA, IPF1, NFAT, CDP, AIRE and OCT1. However, the binding sites for particular sets of transcription factors (e.g. FAC1, POU1F1, STAT1, AR, SRF, LRH) are only enriched in promoters of microvascular EC-restricted genes.

**Figure 7:**
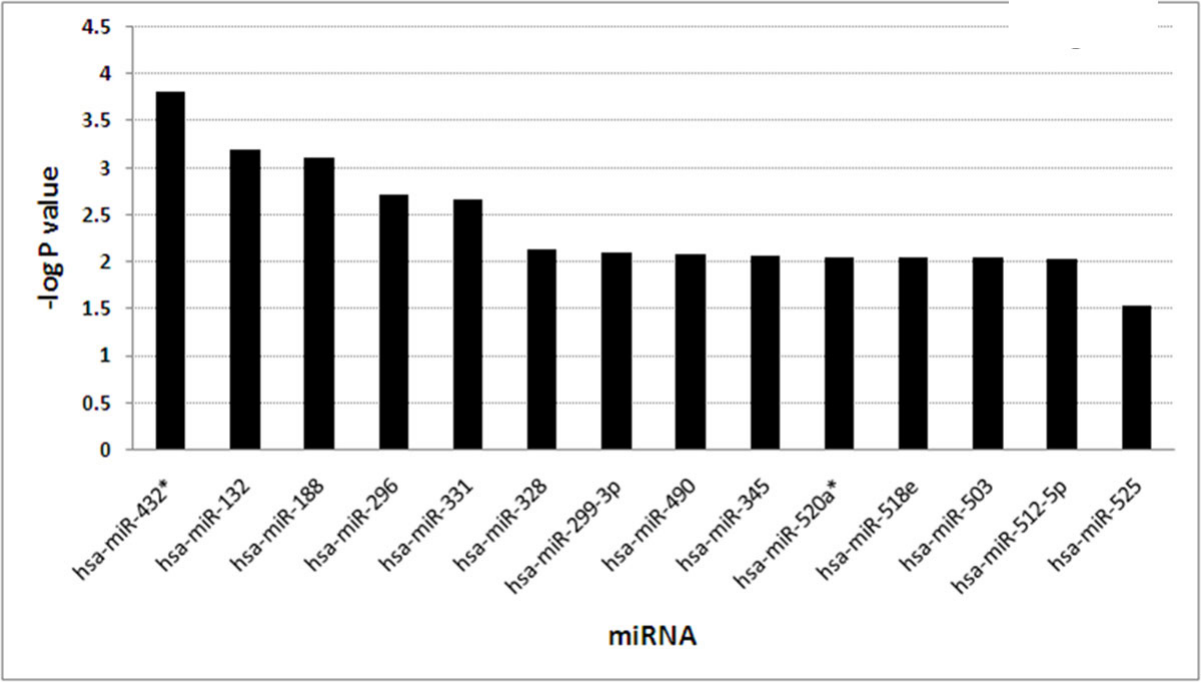
**Regulation analysis of EC-restricted genes in term of MiRNA targets**. The list of the miRNA that are enriched in 3' UTR of EC specific genes. The X-axis represents the miRNA's and Y-axis represents -log P value. The miRNAs from the opposite standard of guided RNA strand are marked with star (*).

Another mechanism by which gene expression can be regulated is through small noncoding RNAs or microRNAs (miRNA). MiRNAs regulate gene expression through translational repression of mRNA by promoting the degradation of mRNA by binding to specific sequences in the untranslated regions of the mRNA. We performed a bioinformatics analysis of the EC-restricted genes in order to identify whether the identified EC-restricted genes are targets of miRNAs. We used *composite regulatory signature database *(CRSD) web tools that take into consideration the sequence match and free energy of binding to predict binding sites [16]. Our analysis identified 31 miRNA binding sites that are significantly enriched (P value < 0.05) in the UTR of the EC-restricted genes [Figure 7]. Mir-432, Mir-188, and Mir-331 target each have putative binding sites in the 3' UTR of >8 EC-restricted genes. A summary of the miRNA binding sites for EC-restricted genes is provided in Table 2. Additionally details of miRNA Binding sites along with target and reference sequences are provided in Additional File 2.

**Table 2:** List of significantly enriched miRNAs binding sites.

microRNA	Hits	P-Value	FDR	**Gene Symbol**,
hsa-miR-432*	11	1.53E-04	0.035458796	PALMD,RAPGEF5,LOC90139,MYLK2, CETP,TIE1,GLCE,VWF,ROBO4,KIAA1274, PDE2A,

hsa-miR-188	10	7.60E-04	0.058786382	CDH5,EGFL7,CXorf36,,RNASE1,SEMA6B, ESAM,RGS3,ROBO4,HYAL2,

hsa-miR-132	6	6.39E-04	0.074133993	IL1RL1,CXorf36,LOC90139,SLC29A1, IPO11,LOC116441,

hsa-miR-331	9	0.002161	0.10028083	TXNRD2,LOC90139,FLJ22746,BCL6B, SEMA6B,ESAM,KIAA1274,TBX1,ICAM2,

hsa-miR-296	9	0.001918	0.111262412	ARHGEF15,CDH5,APLN,RNASE1, SEMA6B,ESAM,ROBO4,CGNL1,

hsa-miR-512-5p	8	0.00932	0.166330341	APLN,RAPGEF5,BCL6B,SLCO2A1, LOC400451,KDR,ROBO4,FLJ46061,

hsa-miR-503	8	0.008909	0.172244646	APLN,LOC90139,ESAM,MGC20262, VWF,ROBO4,ZDHHC14,HYAL2,

hsa-miR-518e	5	0.008905	0.187818163	LAMP3,NOTCH4,BCL6B,GLCE,SEMA6B,

hsa-miR-520a*	8	0.008722	0.202353355	EGFL7,FLJ10241,APLN,ABCA4, HSD17B2,SHANK3,ESAM,CGNL1,

hsa-miR-345	8	0.008564	0.220764545	FLJ10241,RAPGEF5,MYLK2,ADCY4, PLSCR4,GJA4,RGS3,PDE2A,

hsa-miR-490	8	0.008331	0.241605345	CDH5,ESAM,MGC20262,ROBO4, FLJ46061,MOV10L1,CGNL1,ICAM2,

hsa-miR-299-3p	5	0.007968	0.264095479	APLN,TNFRSF11A,TIE1, PECAM1,ROBO4,

hsa-miR-328	8	0.007307	0.282532121	EGFL7,CLDN5,CXorf36,LOC90139,SEMA6B, RGS3,ROBO4,KIAA1274,

hsa-miR-525	7	0.029061	0.293137643	EGFL7,FLJ10241,RAPGEF5,NOTCH4, FLJ22746,ESAM,FLJ46061,

hsa-miR-337	7	0.027316	0.301772827	RAPGEF5,FLJ22746,SHANK3, LOC400451,PLSCR4,KIAA1274,

The miRNAs that are expressed at relatively low level as compared to miRNA from opposite/guided standard are marked with star (*).

### Expression pattern of EC-restricted genes in tissues

**Figure 8:**
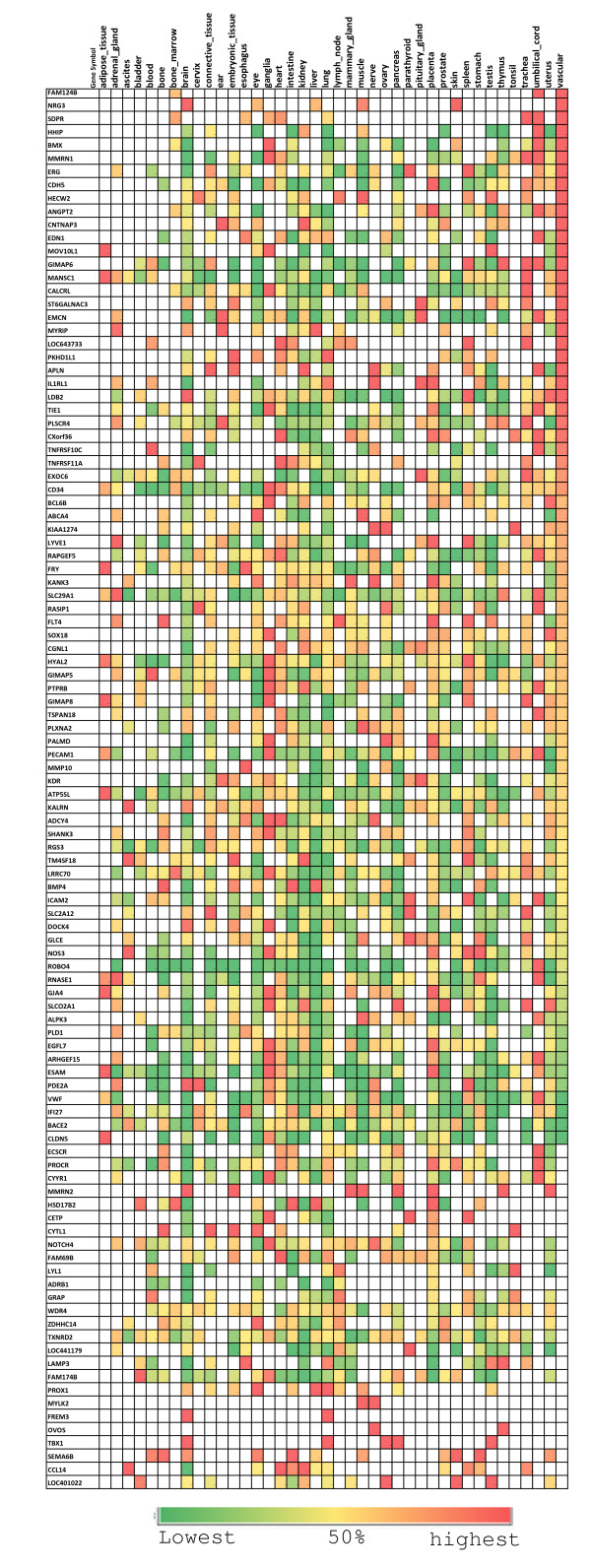
**Relative normalized expression levels of EC-restricted genes in normal tissues**. The expression level is expressed as relative percentage of expression in different tissues with red, yellow and green color denoting higher, median and lower expression levels respectively. The rows represent each gene and columns represent each normal tissue type.

A better understanding of how the EC-restricted genes are expressed in different tissues can help to define their function and potential use as disease biomarkers. Relative expression of the EC-restricted genes in several normal tissues was obtained using the Source databases http://source.stanford.edu. In the source database the normalized gene expression represents the relative expression level of a gene in different tissues. The colorogram depicting the percentage of relative expression of each gene is shown in Figure 8. The analysis demonstrates that most of the endothelial restricted genes have preferential expression in vascular tissues. In particular MMRN1, BMX, ANGPT2 and CDH5 demonstrate high expression levels in vascular tissues. VWF, TIE1, ROBO4 and ECSCR have very high expression levels in umbilical cord tissue (Table 3). These results strengthen our finding that these genes have relatively high expression levels in vascular related tissues.

**Table 3:** Normalized Expression Level of top endothelial restricted genes obtained from the Source database.

Gene Symbol	Rank Score	Normalized Expression
MMRN1	11157.50625	Vascular (29.44%) Umbilical cord (16.7%)

CLDN5	5933.421875	Adipose (66.3%)

VWF	4772.862931	Umbilical cord (43.9%)

BMX	4447.913846	Vascular (56.1%), Umbilical cord (10.1%), Ganglia (11.8%)

ANGPT2	1412.88625	Vascular (19.2%), Umbilical cord (16.3%), Placenta (12.5%)

GJA4	1284.435	Adipose (24.4%), Placenta (10.8%), Ganglia (9.3%)

CDH5	1115.751864	Vascular (19.6%), Placenta (29.4%)

TIE1	1107.308411	Umbilical cord (16.0%), Ganglia (22.2%)

ROBO4	975.358913	Umbilical cord (82.4%)

ECSCR	646.6127586	Umbilical cord (44.6%)

The relative expression level of the genes in different tissues is expression as percentage. The gene expression data for generating the normalized expression level was DbEST database of normal tissues at NCBI.

To further explore whether any of the EC-restricted genes have specific expression in particular tissues, we obtained the immunohistochemistry data for 61 out of the 109 EC-restricted genes. The majority of the EC-restricted genes demonstrate a ubiquitous expression in different normal tissues (Additional File 3). A small subset of the genes show a restricted expression pattern in normal tissues. For example, VWF and ICAM2 are enriched in soft tissues. BMX, one of the top ranked endothelial restricted genes has preferential expression in the epididymis. CLDN5 is preferentially expressed in glandular cells of various body tissues. Interestingly, about 85% of genes depict moderate to high levels of expression in soft tissues.

## Discussion

The results of our study demonstrate that of over 43,000 transcripts evaluated, only 152 appear to be highly restricted to the endothelium. Several of the genes identified have previously been reported to exhibit an EC-restricted expression pattern and have known functions in ECs. Examples of these genes include angiopoietin-2, von Willebrand's Factor (vWF), EC nitric oxide synthase (eNOS), and Pecam-1 (CD31). The pathways, and GO categories of the identified genes support a role for these genes in vascular development, angiogenesis, and EC function.

Although several of the EC-restricted genes have previously been shown to contribute to the regulation of normal EC function, many others have not been characterized as having a particular role in EC. The genes identified as being EC-restricted fall into several categories, including proteins involved in transcriptional regulation, cell adhesion, signal transduction, and intracellular trafficking. The determination that these genes are enriched in ECs may lead to future studies that define their specific role in regulating EC function.

The endothelium is known to play an important role in a number of human diseases, and so it was not a surprise that alterations in the expression of these genes are associated with a number of cardiovascular disorders. Mutations or alterations in the expression of several of the genes listed have been shown to be associated with the development of hypertension. For example, mutations in the eNOS gene have been linked to patients with essential hypertension [21-23]. Similar associations have been observed with mutations in the endothelin-1 gene [24,25]. More recent studies point toward a link between obesity and hypertension. There has been particular interest at understanding the role of adipocytokines and their receptors in the development of hypertension. Previous studies have suggested a causal link between leptin levels in obese patients and the development of hypertension [26]. A more recently discovered adipocytokine, apelin, is predominantly expressed in the ECs of the heart and support a role for apelin in the development of hypertension and cardiac hypertrophy [27].

The endothelium is known to play an important paracrine role with respect to cardiac function and development. The TGFbeta family member cytokine, bone morphogenetic protein-4 (BMP-4), is known to play an important role during cardiac development [28]. Increased expression of BMP-4 may similarly be reflective of a state of EC dysfunction. Exposure of ECs to BMP-4 promotes ROS generation [29]. BMP-4 expression is increased in EC exposed to abnormal or unstable flow, compared to regions of laminar shear flow [30]. Venous and microvessel ECs exposed to BMP-4 rapidly undergo apoptosis [31]. These results suggest the possibility that BMP-4 could be a possible therapeutic target in the setting of heart failure to improve or reverse EC dysfunction.

The functional and structural integrity of the central nervous system depends on tightly controlled coupling between neural activity and cerebral blood flow. This requires the close interaction of neuronal cells and vascular cells in a complex that is known as the neurovascular unit. Recent experimental evidence suggest that dysfunction of the neurovascular unit may be an early event in Alzheimer's disease. Studies in transgenic mice overexpressing the amyloid precursor protein (APP) exhibit abnormalities in blood flow in response to functional hyperemia prior to the development of amyloid plaques or vascular amyloid [32]. Administration of soluble amyloid beta protein results in vasoconstriction, EC dysfunction and a reduction in CBF. One of the main mechanisms by which EC dysfunction occurs is through inactivation or reduced function of EC nitric oxide synthase (eNOS). Amyloid beta also induces the production of reactive oxygen species, alteration in the expression of tight junction proteins, and an increased rate of EC apoptosis [33]. In the brain tissue samples of patients with AD, we observed a significant increase in the expression of selected adherens and tight junction proteins including VE-cadherin, claudin-5, and connexin 37 (GJA4). Systemic administration of the amyloid beta peptide 1-42 to rats is associated with alterations in the expression and cellular localization of several tight junction proteins [33]. Another EC-restricted gene found to be significantly upregulated in the AD brain tissue samples is von Willebrand's Factor (vWF). Increased levels of vWF promote blood clotting. Increased vWF has been found in heme-rich deposits (HRDs) in patients with dementia [34]. HRDs are also rich in fibrinogen, collagen IV, and red blood cells, and are thought to be the residua of capillary bleeds, or microhemorrhages. In patients with acute ischemic stroke and vascular dementia, vWF levels have also been shown to be increased [35].

Our analysis of potential transcription factors that might be involved in regulating the expression of the identified EC-restricted genes, based on conserved binding sites in the regulatory regions of these genes led to the identification of several classes of transcription factors. Most of these transcription factors have not previously been described as playing a major role in the regulation of EC-restricted genes with some exceptions. Members of the ETS and GATA transcription factor families have been shown to regulate a number of endothelial genes including vWF, VE-cadherin, and Tie1 [36-38]. Interestingly, several conserved binding sites were identified only in the regulatory regions of the microvascular ECs suggesting that members of these transcription factor families may play a unique role in determining endothelial gene expression in microvessels.

Over the past several years a role for microRNAs has been demonstrated to play a role in regulating EC gene expression, function, and in the process of angiogenesis. Although most of the miRNAs we identified have not been described for their roles in regulating EC-restricted genes, a few have. For example, hsa-miR-296 has recently been shown to play a regulatory role in angiogenesis (39). Angiogenic factors can increase the expression of hsa-miR-296. Down regulation of hsa-miR-296 in ECs inhibits angiogenic responses in cultured ECs. Furthermore, inhibition of hsa-miR-296 with antagomirs reduced angiogenesis in tumor xenografts in vivo. Similarly, hsa-miR-328 has been implicated in the regulation of CD44 [39]. CD44 regulates a wide variety or processes including angiogenesis and inflammation. The fact that only a small subset of the more than 700 microRNAs has thus far been shown to regulate EC-restricted genes or play a role in regulating EC function suggests that several additional members, including those we have identified, may well also play a role in regulating the expression of selected EC-restricted genes or EC function.

We recognize that there are potential limitations of our study. First, the study used expression-profiling data based on RNA obtained from human tissues or cells. Although several of the genes identified are known to be vascular-specific, the newly identified genes will ultimately need further validation as to the true extent of their EC specificity, at the level of protein and/or RNA both in cells and tissues, and to validate their EC-restricted pattern within the identified tissues.

## Conclusion

Our study validates the existence of a finite number of endothelial-restricted genes most of which are ubiquitously expressed. Several of these are restricted to cells of microvascular origin. Although several of the genes are known to play important roles in endothelial function, the exact functional role of many others in endothelial cells remains to be defined. We hope that our study provides an initial catalogue of EC-restricted genes that can lead to further studies that either link alterations in the expression of these genes to a variety of human diseases via their role as biomarkers or are ultimately shown to play a causal role in the pathogenesis of the particular human diseases.

## Authors' contributions

MB contributed to the overall experimental design, bioinformatics analysis and writing of manuscript. LY contributed in cell culture and RNA extraction. DBK contributed in isolation of B cell and T cells from donors Blood. HHO contributed in statistical analysis. TAL contributed in analysis of result and writing of manuscript. PO contributed to the overall design of the experiments and writing of the manuscript. All authors have read and approved the final manuscript.

## Supplementary Material

Additional file 1 **Nucleotide sequence of primers used for RT-PCR to validate expression pattern of selected EC-restricted genes**. Click here for file

Additional file 2 **Summary of miRNA Binding sites along with target and reference sequences**. Click here for file

Additional file 3 **Immunohistochemistry based expression level of genes in different tissues**. Rows represent the different tissues and columns represent the different EC-restricted genes. The expression level is shown in four color circle scheme i) Red represents strong expression ii) Orange represents moderate expression, iii) Yellow represents weak expression, iv) White represents no detectable expression and Black represents no representative images. The data was obtained from human protein atlas database. Click here for file
