# Supplementary material for: Bioinformatic identification and characterization of human endothelial cell-restricted genes
Source: BMC Genomics. 2010 May 28;11:342. doi: 10.1186/1471-2164-11-342 (PMC2887814; doi:10.1186/1471-2164-11-342)
Supplement: Additional file 1 — Nucleotide sequence of primers used for RT-PCR to validate expression pattern of selected EC-restricted genes. [file 1471-2164-11-342-S1.PDF]

Table S1: The sequence of the Primers for Q RT-PCR analysis.

Primer name	Sequence
VE-Cad-F	GAACCCAAGATGTGGCCTTTAG
VE-Cad-R	GATGTGACAACAGCGAGGTGTAA
ERG-F	CAGCAGGATTGGCTGTCTCA
ERG-R	CATTCACCTGGCTAGGGTTACAT
ROBO4-F	ATGGTGGAAAGATGGGAAACC
ROBO4-R	CTTCGTCACTCTTCTCTGCTCTTG
VWF-F	GTCGAGCTGCACAGTGACATG
VWF-R	GCACCATAAACGTTGACTTCCA
TIE1-F	CACGACCATGACGGCGAAT
TIE1-R	CGGCAGCCTGATATGCCTG
APLN-F	GTGAAGTGGGTGGAGCATCA
APLN-R	GGCACACTAAGGCAAGAGAAGTG
CLDN5-F	CTCTGCTGGTTCGCCAACAT
CLDN5-R	CAGCTCGTACTTCTGCGACA
ICAM2-F	ATCTGTCCTGCTCTGCTTCATCT
ICAM2-R	CGTAGGTGCCCATCCGC
BMX-F	GACGCCTGTAGAGAGACAGTA
BMX-R	CGTCCACGAAGAACCCACTAT
EMCN-F	GTTCTGGTGGGTTTGTACCGAAT
EMCN-R	TCAGACTGAGGTTGATCATTTCCA
MMRN-F	GGCATTGGGCTTAACAACAGT
MMRN-R	AGTTTGATTTCTCACACCCTCAG
GJA4-F	GACCAGGTCCGAGAGCACT
GJA4-R	CCGTGTTACACTCGAAATCTGA
