# Supplementary material for: Bioinformatic identification and characterization of human endothelial cell-restricted genes
Source: BMC Genomics. 2010 May 28;11:342. doi: 10.1186/1471-2164-11-342 (PMC2887814; doi:10.1186/1471-2164-11-342)
Supplement: Additional file 2 — Summary of miRNA Binding sites along with target and reference sequences. [file 1471-2164-11-342-S2.PDF]

Table S2: Summary of miRNA Targets Binding sites analysis along with query and reference sequences. miRNAs that are expressed at relatively low level as compared to miRNA from opposite/guided standard a marked with star (*).

<table>
<tr><th>miRNA</th><th>Alignment</th><th>Gene Symbol</th><th>Energy</th></tr>
<tr><td rowspan="11">hsa-miR-432*</td><td>Query: 3' UCUGUAC---CUCCUCGGUAGGuc 5'
|||:: | ||||| ||||||
Ref: 5' AGATGGGACTGAGGATCCATCCtt 3'</td><td>PALMD</td><td>-22.84</td></tr>
<tr><td>Query: 3' ucUGUACCUCCUCGG-UAGGUC 5'
| |||| | |||| ||||||
Ref: 5' ctAAATGGCGCAGCCAATCCAG 3'</td><td>RAPGEF5</td><td>-22.56</td></tr>
<tr><td>Query: 3' UCUGUAC-CUCC---UCGGUAGGUC 5'
||||| | |||| ||:|| ||||
Ref: 5' AGACAAGAGAGGGCAAGTCAGCCAG 3'</td><td>LOC90139</td><td>-24.93</td></tr>
<tr><td>Query: 3' uCUGUACCUCCUCGGUAGGUC 5'
| |||||:| |||| |||||
Ref: 5' gGCCATGGGGCAGCC-TCCAG 3'</td><td>MYLK</td><td>-28.26</td></tr>
<tr><td>Query: 3' uCUGUAC-CUC-------CUC-GGUAGGUC 5'
||| || ||| ||| ||||||||
Ref: 5' tGAC-TGAGAGCAGCTCCGAGTCCATCCAG 3'</td><td>CETP</td><td>-24.84</td></tr>
<tr><td>Query: 3' UCUGUACCUCCUCGGUAGGUC 5'
||:| | ||| |||||||||
Ref: 5' AGGCCT-GAGCTGCCATCCAG 3'</td><td>TIE1</td><td>-25.91</td></tr>
<tr><td>Query: 3' ucuGUACCUCCUCGGUAGGUC 5'
||| | || ||||||||
Ref: 5' cttCATCCACGAACCATCCAG 3'</td><td>GLCE</td><td>-17.77</td></tr>
<tr><td>Query: 3' uCUGUACCU-CCUC-GGUAGGUC 5'
|||| ||| |||| |||| |||
Ref: 5' cGACACGGACGGAGCCCATGCAG 3'</td><td>VWF</td><td>-27.82</td></tr>
<tr><td>Query: 3' UCUGUACCUCC--UCG--GUAGGUC 5'
||||||||||| ||| || ||||
Ref: 5' AGACATGGAGGTCAGCCACACCCAG 3'</td><td>ROBO4</td><td>-34.25</td></tr>
<tr><td>Query: 3' uCUGUACCUC---C---UCGGUAGGuc 5'
|||| |||| | ||||||||
Ref: 5' gGACAGGGAGACAGCACAGCCATCCct 3'</td><td>KIAA1274</td><td>-23.2</td></tr>
<tr><td>Query: 3' UCUGUACCUCCUCGGUAGGUc 5'
||::||| | |||||| |||
Ref: 5' AGGTATGAATGAGCCAGCCAt 3'</td><td>PDE2A</td><td>-20.07</td></tr>
<tr><td rowspan="6">hsa-miR-132</td><td>Query: 3' gCUGGUACCGACAUCUGACAAu 5'
||:| | |||||||||||
Ref: 5' tGATCTT---TGTAGACTGTTc 3'</td><td>IL1RL1</td><td>-22.12</td></tr>
<tr><td>Query: 3' GCUGGUACCGACAUCUGACAAU 5'
|||||:| || |||||||||
Ref: 5' CGACCGT--CTTCAGACTGTTA 3'</td><td>CXorf36</td><td>-24.85</td></tr>
<tr><td>Query: 3' gCUGGU-ACCGAC-AUCUGACAAu 5'
| ||| ||| | |:|||||||
Ref: 5' gGTCCACTGGAGGATGGACTGTTc 3'</td><td>LOC90139</td><td>-21.72</td></tr>
<tr><td>Query: 3' GCUGGUACCGACA-UCUGACAau 5'
|| ||:|| |||| ||:||||
Ref: 5' CGTCCGTGTCTGTCAGGCTGTct 3'</td><td>SLC29A1</td><td>-25.41</td></tr>
<tr><td>Query: 3' gcugGUACCGACA---UCUGACAAu 5'
|:||||| | ||||||||
Ref: 5' cccaCGTGGCTATGACAGACTGTTt 3'</td><td>IPO11</td><td>-22.5</td></tr>
<tr><td>Query: 3' gcUGGUA-CCGAC--AUCUGACAAU 5'
|||:| |:|| |:||||||||
Ref: 5' gcACCGTACGTTGAATGGACTGTTA 3'</td><td>LOC116441</td><td>-22.1</td></tr>
<tr><td rowspan="10">hsa-miR-188</td><td>Query: 3' uGGGAG-GUGGUACGUUCCCUAc 5'
||||| ||| ||||||||||
Ref: 5' cCCCTCACAC--TGCAAGGGATt 3'</td><td>CDH5</td><td>-29.85</td></tr>
<tr><td>Query: 3' uGGGAGG--UGGUA-CGUUCCCUAC 5'
|:| || ||| | ||| ||||||
Ref: 5' gCTCCCCAGACCCTGGCATGGGATG 3'</td><td>EGFL7</td><td>-25.88</td></tr>
<tr><td>Query: 3' ugGGAGGUGGUACGUUCCCUac 5'
||||||:||| ||||||
Ref: 5' tgCCTCCATCAT-AAAGGGAgg 3'</td><td>CXorf36</td><td>-28.93</td></tr>
<tr><td>Query: 3' ugggagGUGGU-AC--GUUCCCUAC 5'
||||| || :||||||||
Ref: 5' gaagaaCACCAGTGTTTAAGGGATG 3'</td><td>Hs.382865</td><td>-23.85</td></tr>
<tr><td>Query: 3' UGGGA-GGUGGUAC----GUUCCCuac 5'
||||| :||||||| :|||||
Ref: 5' ACCCTATCACCATGTTCTTAAGGGggg 3'</td><td>RNASE1</td><td>-31.37</td></tr>
<tr><td>Query: 3' uGGGAGGUGGUACG-UUCCCUac 5'
||| ||||: ||| :|||||
Ref: 5' cCCCCCCACTCTGCAGAGGGAag 3'</td><td>SEMA6B</td><td>-27.6</td></tr>
<tr><td>Query: 3' UGGGAGGUGGUACGUUCCCUac 5'
|||||||::: | |:|||||
Ref: 5' ACCCTCCGTTTTCCGAGGGAag 3'</td><td>ESAM</td><td>-29.83</td></tr>
<tr><td>Query: 3' UGGGAGGUGGU--ACGUUCCCUAc 5'
||| |:| ||| | ||:|||||
Ref: 5' ACCATTCTCCAGCTTCAGGGGATa 3'</td><td>RGS3</td><td>-25.03</td></tr>
<tr><td>Query: 3' uggGAGGUGGUAC-GUUCCCUAc 5'
|||: ||||| |||||| |
Ref: 5' cgtCTCTCCCATGCCAAGGGCTc 3'</td><td>ROBO4</td><td>-25.17</td></tr>
<tr><td>Query: 3' ugGGAGGUGGUACGUUCCCUAc 5'
||||:|||| |||||| |
Ref: 5' ggCCTCTACCA--CAAGGGCTc 3'</td><td>HYAL2</td><td>-26.55</td></tr>
</table>

<table>
<tr><td rowspan="10">hsa-miR-331</td><td>Query: 3' aaGAUCCUAUCCGGGUCCCCg 5'
  | :|||| |||:||||||
Ref: 5' caCAGGGAT-GGCTCAGGGGa 3'</td><td>TXNRD2</td><td>-30.36</td></tr>
<tr><td>Query: 3' aaGAUCCU-AUCCGGGUCCCCg 5'
  | |||| ||| |||||||
Ref: 5' ggCCAGGAGAAGGGCCAGGGGa 3'</td><td>LOC90139</td><td>-29.41</td></tr>
<tr><td>Query: 3' aagauccuaUCCGGGUCCCCG 5'
         | ||||||||||
Ref: 5' aagtcatcaAAGCCCAGGGGC 3'</td><td>LOC90139</td><td>-24.97</td></tr>
<tr><td>Query: 3' aaGAUCC---UAUCC-GGGUCCCCG 5'
  | ||| | ||| |||||||||
Ref: 5' aaCCAGGGCAAGAGGTCCCAGGGGC 3'</td><td>FLJ22746</td><td>-32.2</td></tr>
<tr><td>Query: 3' AAGA-UCCUAUC-CG---GGUCCCCG 5'
|||| |||| || || |:|||||
Ref: 5' TTCTGAGGAGAGAGCTAGCTAGGGGC 3'</td><td>BCL6B</td><td>-26.36</td></tr>
<tr><td>Query: 3' AAGAUCCUAUCCGGGUCCCCg 5'
||| | || ||||||:||||
Ref: 5' TTCCA-GA-AGGCCCGGGGGt 3'</td><td>SEMA6B</td><td>-28.13</td></tr>
<tr><td>Query: 3' aaGAUCCUAUCCG--GGUCCCCG 5'
  || | |||| |||||||
Ref: 5' ccCTTG---AGGCCTCCAGGGGC 3'</td><td>ESAM</td><td>-24.11</td></tr>
<tr><td>Query: 3' aagaUCCUAUC-CG--GGUCCCCg 5'
    |||| || || |||||||
Ref: 5' ggggAGGAAAGAGCTTCCAGGGGg 3'</td><td>KIAA1274</td><td>-24.36</td></tr>
<tr><td>Query: 3' aagauccuaUCCGGGUCCCCG 5'
         || |||||||||
Ref: 5' cgttcccccAGCCCCAGGGGC 3'</td><td>TBX1</td><td>-23.47</td></tr>
<tr><td>Query: 3' aagaUCCUA-UCCG---GGUCCCCG 5'
    |||:| |||| |||||||
Ref: 5' gaagAGGGTGAGGCTGTCCAGGGGC 3'</td><td>ICAM2</td><td>-30.88</td></tr>
<tr><td rowspan="11">hsa-miR-296</td><td>Query: 3' UGU--CCUAACUCCCCCCCGGGa 5'
||| || | | |||||||||
Ref: 5' ACAGTGGCTGGCAGGGGGGCCCa 3'</td><td>ARHGEF15</td><td>-32.3</td></tr>
<tr><td>Query: 3' uGUCCUAACUCC-CCCCCGGGa 5'
 || || |||| |||||||
Ref: 5' tCAAGAGGGAGGAAGGGGCCCc 3'</td><td>CDH5</td><td>-28.27</td></tr>
<tr><td>Query: 3' uGUCCUAACUCCCCCCCGGGa 5'
 ||||| ||| |||||||
Ref: 5' gCAGGACTGA--AGGGGCCCc 3'</td><td>APLN</td><td>-28.27</td></tr>
<tr><td>Query: 3' uguccuAACUCCCCCCCGGGA 5'
      || ||||||||||||
Ref: 5' atgttcTTAAGGGGGGGCCCT 3'</td><td>RNASE1</td><td>-30.96</td></tr>
<tr><td>Query: 3' uguccuaacUCCCCCCCGGGa 5'
          |||||||||||
Ref: 5' ---------AGGGGGGGCCCc 3'</td><td>Hs.483538</td><td>-28.27</td></tr>
<tr><td>Query: 3' uguccuaaCUC-CCCCCCGGGa 5'
        ||| |||||||||
Ref: 5' cacccgcgGAGTGGGGGGCCCc 3'</td><td>SEMA6B</td><td>-29.29</td></tr>
<tr><td>Query: 3' uGUC-CUAACUCCC-CCCCGGGa 5'
 |:| || | ||| |||||||
Ref: 5' gCGGCGAAGGTGGGTGGGGCCCc 3'</td><td>SEMA6B</td><td>-31.19</td></tr>
<tr><td>Query: 3' uGUCCUAACUCCC-----CC-CCGGGa 5'
 |:||| |||||| || |||||
Ref: 5' tCGGGAGTGAGGGACCCTGGCGGCCCg 3'</td><td>ESAM</td><td>-31.9</td></tr>
<tr><td>Query: 3' uGUCCUAA----CUCCC--CCCCGGGa 5'
 ||||| | ||||| |||||||
Ref: 5' gCAGGACTGGAGGAGGGGTGGGGCCCa 3'</td><td>ROBO4</td><td>-33.53</td></tr>
<tr><td>Query: 3' UGUCC--UAACUCCC---CCCCGGGa 5'
|:||| : || ||| |||||||
Ref: 5' ATAGGCAGATGTGGGTATGGGGCCCa 3'</td><td>CGNL1</td><td>-26.72</td></tr>
<tr><td>Query: 3' ugUC-CUAACUCC---CCCCCGGga 5'
  || |:|||||| |||||||
Ref: 5' ggAGTGGTTGAGGTCTGGGGGCCag 3'</td><td>CGNL1</td><td>-31.44</td></tr>
<tr><td rowspan="7">hsa-miR-512-5p</td><td>Query: 3' cuUUCACGGGA----GUUCCGACUCac 5'
  |||||||:| |:||||||:|
Ref: 5' tgAAGTGCCTTTTCCCGAGGCTGGGgc 3'</td><td>APLN</td><td>-31.98</td></tr>
<tr><td>Query: 3' cUUUCACGGGAGUUCCGACUCac 5'
 |:| | |||||| |||||:|
Ref: 5' cAGACTTCCCTCACGGCTGGGct 3'</td><td>RAPGEF5</td><td>-25.2</td></tr>
<tr><td>Query: 3' CUU-UCACG--GGAGUUCCGACUCAC 5'
||| || |: |||| |||||:|||
Ref: 5' GAACAGGGTTGCCTCTTGGCTGGGTG 3'</td><td>BCL6B</td><td>-29.43</td></tr>
<tr><td>Query: 3' CUUUC---ACGGGAGUUCCGACUCAc 5'
|||:| ||||:||| |||||||
Ref: 5' GAAGGCACTGCCTTCA--GCTGAGTt 3'</td><td>SLCO2A1</td><td>-24.81</td></tr>
<tr><td>Query: 3' cUUUCACGGGAGUUCCGACUCAC 5'
 | :|||: ||||:| |||||||
Ref: 5' cACGGTGTACTCAGGCCTGAGTG 3'</td><td>LOC400451</td><td>-27.64</td></tr>
<tr><td>Query: 3' CUUUCACGGGAGUUCCGACUCAc 5'
|:||| ::: ||:||||||||
Ref: 5' GGAAGGATTTGCAGGGCTGAGTc 3'</td><td>KDR</td><td>-25.09</td></tr>
<tr><td>Query: 3' cUUUCACGGGAGUUCCGACUCAc 5'
 |||| | :|||||||||:||</td><td>ROBO4</td><td>-23.84</td></tr>
</table>

<table>
<tr><td></td><td>Ref: 5' cAAAGGG--TTCAAGGCTGGGTc 3'</td><td></td><td></td></tr>
<tr><td></td><td>Query: 3' cuuucaCGGGAGUUCCGACUCac 5'
||||| :||||||||
Ref: 5' gccatcGCCCTGGAGGCTGAGca 3'</td><td>FLJ46061</td><td>-28.14</td></tr>
<tr><td rowspan="9">hsa-miR-503</td><td>Query: 3' GACGUCUUGACAAGGGCGACGAu 5'
|||| | ||| | |:|||||
Ref: 5' CTGCTG--CTGCTGCTGCTGCTg 3'</td><td>APLN</td><td>-25.74</td></tr>
<tr><td>Query: 3' GAC-GUCUU---GACAAGGGCGACGau 5'
| | ||||| |||| ||:||||
Ref: 5' CAGCCAGAAGCTCTGTGCCTGCTGCaa 3'</td><td>LOC90139</td><td>-24.89</td></tr>
<tr><td>Query: 3' GACGUCUUGACAAGGGCGACGau 5'
|||| |: || ||||||| ||
Ref: 5' CTGC-GGCCTCTTCCCGCGGCgt 3'</td><td>ESAM</td><td>-24.81</td></tr>
<tr><td>Query: 3' GACGUCUUGACAAGG-GCGACgau 5'
||||: | |||| || |||||
Ref: 5' CTGCGCACCTGTACCACGCTGagc 3'</td><td>MGC20262</td><td>-24.5</td></tr>
<tr><td>Query: 3' gACGUCUUG---ACAAGGGCGACGAu 5'
|||||:|| || |||:||||||
Ref: 5' gTGCAGGACCAGTGCTCCTGCTGCTc 3'</td><td>VWF</td><td>-30.72</td></tr>
<tr><td>Query: 3' GACGUCUUGA------CAAGGGCGACGAu 5'
|||||| || || ||:||||||
Ref: 5' CTGCAG--CTGCATGGGTGCCTGCTGCTg 3'</td><td>VWF</td><td>-29.88</td></tr>
<tr><td>Query: 3' gaCGUCUUGACAAGGGCGACGAu 5'
|||| ||| ||:||||||
Ref: 5' gaGCAGCCCTG--CCTGCTGCTc 3'</td><td>ROBO4</td><td>-25.42</td></tr>
<tr><td>Query: 3' gACGUCUUGAC--AAGG-GCGACGau 5'
||::|| ||| | || ||||||
Ref: 5' gTGTGGATCTGCATGCCACGCTGCcg 3'</td><td>ZDHHC14</td><td>-23.3</td></tr>
<tr><td>Query: 3' gACGUCU-UGACAAGGGCGACGau 5'
|||||| || || |||||||
Ref: 5' -TGCAGACACACTT-CCGCTGCca 3'</td><td>HYAL2</td><td>-24.58</td></tr>
<tr><td rowspan="5">hsa-miR-518e</td><td>Query: 3' UGUGAGACUUCC------CUUCGCGAAA 5'
|| |||||:||| ||:| |||||
Ref: 5' ACTCTCTGGAGGGAGACTGAGGGGCTTT 3'</td><td>LAMP3</td><td>-27.24</td></tr>
<tr><td>Query: 3' uGUGAGACUUC-CCUUCGCGAAa 5'
|||| ||::| |||||||||
Ref: 5' cCACTGTGGGGATGAAGCGCTTc 3'</td><td>NOTCH4</td><td>-26.27</td></tr>
<tr><td>Query: 3' uguGAGA-CUUCCCU-UCGCGAAa 5'
|||| ||||||| :|:||||
Ref: 5' ttcCTCTAGAAGGGATGGTGCTTg 3'</td><td>BCL6B</td><td>-26.71</td></tr>
<tr><td>Query: 3' uGUGAGACUUCCCUUCGCGAAA 5'
| :||||||||| ||: ||||
Ref: 5' cCCTTCTGAAGGGTAGTCCTTT 3'</td><td>GLCE</td><td>-23.43</td></tr>
<tr><td>Query: 3' uGUGAGAC--UUCCCUUCGCgaaa 5'
||||||| :|||||||||
Ref: 5' cCACTCTGCAGAGGGAAGCGggga 3'</td><td>SEMA6B</td><td>-29.7</td></tr>
<tr><td rowspan="8">hsa-miR-520a*</td><td>Query: 3' uCUUUCAUG---AAGGGA-GACCUC 5'
|||:|||| ||||| ||||||
Ref: 5' gGAAGGTACGAGCTCCCTGCTGGAG 3'</td><td>EGFL7</td><td>-31.27</td></tr>
<tr><td>Query: 3' ucuuuCAUGAAG--GGAGACCUc 5'
||:|||| ||||||||
Ref: 5' tggttGTGCTTCTGCCTCTGGAc 3'</td><td>FLJ10241</td><td>-24.75</td></tr>
<tr><td>Query: 3' UCUUUCAUGAAG-GGAGACCUC 5'
||| | || | :||||||||
Ref: 5' AGATTGGTCTGCTTCTCTGGAG 3'</td><td>APLN</td><td>-21.67</td></tr>
<tr><td>Query: 3' uCUUUCA----UGAAGGGAGACCUc 5'
|||||| || |||||||| |
Ref: 5' tGAAAGTCATGACCTCCCTCTGCAc 3'</td><td>ABCA4</td><td>-20.32</td></tr>
<tr><td>Query: 3' UCUUUCAUGAAGGGAGACCuc 5'
|| ||| |||||:||| ||
Ref: 5' AGCAAGGACTTCTCTCCGGtg 3'</td><td>HSD17B2</td><td>-20.14</td></tr>
<tr><td>Query: 3' UCUUUCAUGAAG-GGAGACCUC 5'
||||: :|| | ||||||||
Ref: 5' AGAAGCCGCTGCAGCTCTGGAG 3'</td><td>SHANK3</td><td>-21.7</td></tr>
<tr><td>Query: 3' UCUUUCAUGAAG-GGAGACCUc 5'
||||| | || | ||||||||
Ref: 5' AGAAA-T-CTGCGCCTCTGGAt 3'</td><td>ESAM</td><td>-18.46</td></tr>
<tr><td>Query: 3' UCUUUCAUGAAGGGA-GACCUC 5'
| ||| | || |||| ||||||
Ref: 5' AAAAATTCCTCCCCTCCTGGAG 3'</td><td>CGNL1</td><td>-20.1</td></tr>
<tr><td rowspan="5">hsa-miR-345</td><td>Query: 3' CG-GGAC--C-UGAUCC-UCAGUCGU 5'
|| |||| | || ||| :|||||||
Ref: 5' GCTCCTGAAGCACCAGGCGGTCAGCA 3'</td><td>FLJ10241</td><td>-24.51</td></tr>
<tr><td>Query: 3' CGGGACCUGAUCCUCAGUCGU 5'
||||| | :||| |||:||||
Ref: 5' GCCCT-GCTTAGAAGTTAGCA 3'</td><td>RAPGEF5</td><td>-26.13</td></tr>
<tr><td>Query: 3' CGGGACCUGAU-CCUCAGUCGU 5'
|||: ||: | ||:|||||||
Ref: 5' GCCTCAGATGATGGGGTCAGCA 3'</td><td>MYLK2</td><td>-26.11</td></tr>
<tr><td>Query: 3' cGGGAC-CUGA-UCCUCAGUCGU 5'
|| | |||| ||||| |||||
Ref: 5' tCCAAGTGACTGAGGAGACAGCA 3'</td><td>ADCY4</td><td>-25.54</td></tr>
<tr><td>Query: 3' cgggACCUGAUCC-----UCAGUCGU 5'
||||:|:|| ||||||||</td><td>PLSCR4</td><td>-27.77</td></tr>
</table>

<table>
<tr><td rowspan="4"></td><td>Ref: 5' aagtTGGATTGGGCTTACAGTCAGCA 3'</td><td></td><td></td></tr>
<tr><td>Query: 3' cgGGACCUG-AUC---CUCAGUCGU 5'
|||| :| || |||||||||
Ref: 5' tgCCTGAGCACAGACAGAGTCAGCA 3'</td><td>GJA4</td><td>-23.92</td></tr>
<tr><td>Query: 3' CGG--GACCUGAUCCUCAGUCGu 5'
||| |||||||| || ||||
Ref: 5' GCCTTCTGGACTA--AGGCAGCc 3'</td><td>RGS3</td><td>-22.12</td></tr>
<tr><td>Query: 3' CGGGACCUGA-UCCUCAGUCGu 5'
| || ||||| |:||||||
Ref: 5' GGCCGGGACTCTTGGGTCAGCc 3'</td><td>PDE2A</td><td>-25.98</td></tr>
<tr><td rowspan="8">hsa-miR-490</td><td>Query: 3' GUC-GUACCUCA--G---GAGGUCCAAc 5'
||| | |||: | | |||||||||
Ref: 5' CAGCCTTGGGATAGCAAACTCCAGGTTc 3'</td><td>CDH5</td><td>-23.7</td></tr>
<tr><td>Query: 3' GUC--GUACCUC-AGGAGGUCCaac 5'
||| || ||| |||||||||
Ref: 5' CAGACCACAGAGATCCTCCAGGaaa 3'</td><td>ESAM</td><td>-24.5</td></tr>
<tr><td>Query: 3' GUCGUAC-CUCAGGAGGU----CCAAc 5'
|:||| | |||||||||| ||||
Ref: 5' CGGCAGGAGAGTCCTCCAAGGGGGTTt 3'</td><td>MGC20262</td><td>-31.2</td></tr>
<tr><td>Query: 3' GUCGUACCUCAGGA---GGU--CCAAC 5'
||||: ||: |||| ||| |||||
Ref: 5' CAGCGAGGGCTCCTTAGCCAATGGTTG 3'</td><td>ROBO4</td><td>-26.16</td></tr>
<tr><td>Query: 3' gucgUACCUC---AGGAGGUCCAAc 5'
|||||| || |||||||
Ref: 5' gacaATGGAGAGAACCCCCAGGTTc 3'</td><td>FLJ46061</td><td>-22.94</td></tr>
<tr><td>Query: 3' guCGUACCUCAGGAGGUCCAAC 5'
|::|||:| |: ||||||||
Ref: 5' ggGTGTGGGG-CTGCCAGGTTG 3'</td><td>MOV10L1</td><td>-28.1</td></tr>
<tr><td>Query: 3' gUC-GUACCUCAGG------A-GGUCCAac 5'
|| |||||||||| | ||||||
Ref: 5' tAGCCATGGAGTCCCACCTGTGCCAGGTca 3'</td><td>CGNL1</td><td>-29.93</td></tr>
<tr><td>Query: 3' GUCGUACCUCAGGAGGU-CCAAC 5'
|| ||||| || |||| |||||
Ref: 5' CACCATGGTGT--TCCACGGTTG 3'</td><td>ICAM2</td><td>-23.21</td></tr>
<tr><td rowspan="5">hsa-miR-299-3p</td><td>Query: 3' uucgCCA-AAUGGUA-GGGUGUAU 5'
||| | |||| ||||||||
Ref: 5' catgGGTGTCCCCATGCCCACATA 3'</td><td>APLN</td><td>-24.05</td></tr>
<tr><td>Query: 3' uuCGCCAAAUGGUAGGGUGUAU 5'
||||||||:: || |||||
Ref: 5' ctGCGGTTTATTCACCAACATA 3'</td><td>TNFRSF11A</td><td>-21.83</td></tr>
<tr><td>Query: 3' UUCGC-CAAAUGGUAGGGUGuau 5'
|:|:| |||| |||||||||
Ref: 5' AGGTGTGTTTCCCATCCCACtgc 3'</td><td>TIE1</td><td>-27.7</td></tr>
<tr><td>Query: 3' uuCGCCAAAUGGUAGGGUGUAU 5'
|| ||| | : |||||||||
Ref: 5' ggGCTGTTGAATTTCCCACATA 3'</td><td>PECAM1</td><td>-20.07</td></tr>
<tr><td>Query: 3' uucgCCAAAUGGUA-GGGUGUAu 5'
||| | || | |||||||
Ref: 5' cttgGGTGTCCCTTCCCCACATg 3'</td><td>ROBO4</td><td>-20.17</td></tr>
<tr><td rowspan="9">hsa-miR-328</td><td>Query: 3' uGCCUUCCCGUCUCUCCCGGuc 5'
||||||| |||: ||||||
Ref: 5' gCGGAAGGCCAGGCAGGGCCtt 3'</td><td>EGFL7</td><td>-34.66</td></tr>
<tr><td>Query: 3' uGCCUUCC------CGUCUC---UCCCGGuc 5'
||||:|| |||||| ||||||
Ref: 5' tCGGAGGGGCGGATGCAGAGCCCAGGGCCcc 3'</td><td>CLDN5</td><td>-34.48</td></tr>
<tr><td>Query: 3' uGCCUUCCC-GUCUC---UCCC-GGUC 5'
||| |||| | ||| |||| ||||
Ref: 5' tCGGCAGGGCCCGAGCCCAGGGACCAG 3'</td><td>CLDN5</td><td>-32.1</td></tr>
<tr><td>Query: 3' UGCCUUCCCG-UCUCUCCCGGUC 5'
| |||:|||| | ||||| ||||
Ref: 5' AGGGAGGGGCAACAGAGGTCCAG 3'</td><td>CXorf36</td><td>-34.34</td></tr>
<tr><td>Query: 3' ugcCUUCCCGU-CUC-UCCCGGUC 5'
|| || || ||| ||||||||
Ref: 5' agaGATGGCCAGGAGAAGGGCCAG 3'</td><td>LOC90139</td><td>-27.36</td></tr>
<tr><td>Query: 3' uGCCUUCCCGUCUCUCCCGGUc 5'
||| :||| | :|:||||||
Ref: 5' cCGGTGGGGAATGGGGGGCCAc 3'</td><td>SEMA6B</td><td>-29.51</td></tr>
<tr><td>Query: 3' UGCCUUC---CCG--------UCUCUCCCGGUC 5'
||||||| ||| ||||||| ||||
Ref: 5' ACGGAAGCGAGGCCTGGACCAAGAGAGGCCCAG 3'</td><td>RGS3</td><td>-33.5</td></tr>
<tr><td>Query: 3' UGCCU-UCCCGUC--UCUCCCGGUC 5'
| ||| || |||| ||| ||||||
Ref: 5' AGGGACAGTGCAGATAGAAGGCCAG 3'</td><td>ROBO4</td><td>-25.69</td></tr>
<tr><td>Query: 3' uGCCUUCC-CGUCUCUCCCGGUC 5'
||| :|| ||||| | ||||||
Ref: 5' cCGGTGGGAGCAGAAAAGGCCAG 3'</td><td>KIAA1274</td><td>-30.14</td></tr>
<tr><td rowspan="3">hsa-miR-525</td><td>Query: 3' uCUUUCACGU---AGGGA-GACCUC 5'
|||:|| |: ||||| ||||||
Ref: 5' gGAAGGTACGAGCTCCCTGCTGGAG 3'</td><td>EGFL7</td><td>-27.81</td></tr>
<tr><td>Query: 3' ucuuuCACGUAG--GGAGACCUc 5'
|||| || ||||||||
Ref: 5' tggttGTGCTTCTGCCTCTGGAc 3'</td><td>FLJ10241</td><td>-22</td></tr>
<tr><td>Query: 3' UCUUUC-----ACGUAGG-GAGACCUc 5'
||:| | |||| || |||||||</td><td>RAPGEF5</td><td>-21.35</td></tr>
</table>

<table>
<tr><td rowspan="5"></td><td>Query: 3' UCUUUC-----ACGUAGG-GAGACCUc 5'
||:| | |||| || |||||||
Ref: 5' AGGATGGGCCATGCACCCTCTCTGGAt 3'</td><td>RAPGEF5</td><td>-21.35</td></tr>
<tr><td>Query: 3' UCUUUCACGUAGGGAGACCUc 5'
||||: |||| ||||||||
Ref: 5' AGAAGCTGCA--GCTCTGGAa 3'</td><td>NOTCH4</td><td>-20.64</td></tr>
<tr><td>Query: 3' ucuUUCACGUAGGGAGACCUC 5'
|:| |||| :||||||||
Ref: 5' cccAGGGGCAT-TCTCTGGAG 3'</td><td>FLJ22746</td><td>-23.83</td></tr>
<tr><td>Query: 3' UCUUU-CACGUAGGGAGACCUc 5'
||||| |||: ||||||||
Ref: 5' AGAAATCTGCG--CCTCTGGAt 3'</td><td>ESAM</td><td>-20.5</td></tr>
<tr><td>Query: 3' UCUUUCACGUAGGGAGACCuc 5'
||:||| | || ||||||
Ref: 5' AGGAAGGGGCTCACTCTGGcc 3'</td><td>FLJ46061</td><td>-20.64</td></tr>
<tr><td rowspan="7">hsa-miR-337</td><td>Query: 3' UUUCCGUAGUAUAUCCUCGACCu 5'
||:|||||| | | |:|||||
Ref: 5' AAGGGCATCCTCCA-GGGCTGGt 3'</td><td>RAPGEF5</td><td>-26.22</td></tr>
<tr><td>Query: 3' UUUCCGUAGUAUAUCCUCGACCu 5'
|| |||||| | |:||||||||
Ref: 5' AATGGCATC-T-TGGGAGCTGGc 3'</td><td>FLJ22746</td><td>-29.25</td></tr>
<tr><td>Query: 3' uUUCC-GUAGUAUAUCCUCGACCu 5'
|||| |: | | ||||||||
Ref: 5' cAAGGACGACTTCGTGGAGCTGGg 3'</td><td>SHANK3</td><td>-23.99</td></tr>
<tr><td>Query: 3' UUUCCGU-AGUAUAUCCUCGACCu 5'
|| |||| | | |||||||||
Ref: 5' AATGGCAGGAACAAAGGAGCTGGg 3'</td><td>Hs.382865</td><td>-25.32</td></tr>
<tr><td>Query: 3' uUUCCGUAGUAUAU-CCUCGACCU 5'
||| || : || | ||:||||||
Ref: 5' gAAGTCAGTCTAGACGGGGCTGGA 3'</td><td>LOC400451</td><td>-20.92</td></tr>
<tr><td>Query: 3' uUUCCG---UAGU---AUA------UCCUCGACCu 5'
||||| |||| ||| |||||||||
Ref: 5' gAAGGCAGAATCAGACTATCAGGAAAGGAGCTGGc 3'</td><td>PLSCR4</td><td>-26.55</td></tr>
<tr><td>Query: 3' uuUCCGUAGUAUAUCCUCGACCU 5'
|||: || | ||||||||||
Ref: 5' ggAGGTTTC-TCAAGGAGCTGGA 3'</td><td>KIAA1274</td><td>-26.63</td></tr>
</table>
