# Supplementary material for: Bioinformatic identification and characterization of human endothelial cell-restricted genes
Source: BMC Genomics. 2010 May 28;11:342. doi: 10.1186/1471-2164-11-342 (PMC2887814; doi:10.1186/1471-2164-11-342)
Supplement: Additional file 3 — Immunohistochemistry based expression level of genes in different tissues. Rows represent the different tissues and columns represent the different EC-restricted genes. The expression level is shown in four color circle scheme i) Red represents strong expression ii) Orange represents moderate expression, iii) Yellow represents weak expression, iv) White represents no detectable expression and Black represents no representative images. The data was obtained from human protein atlas database. [file 1471-2164-11-342-S3.PDF]

Fig S1

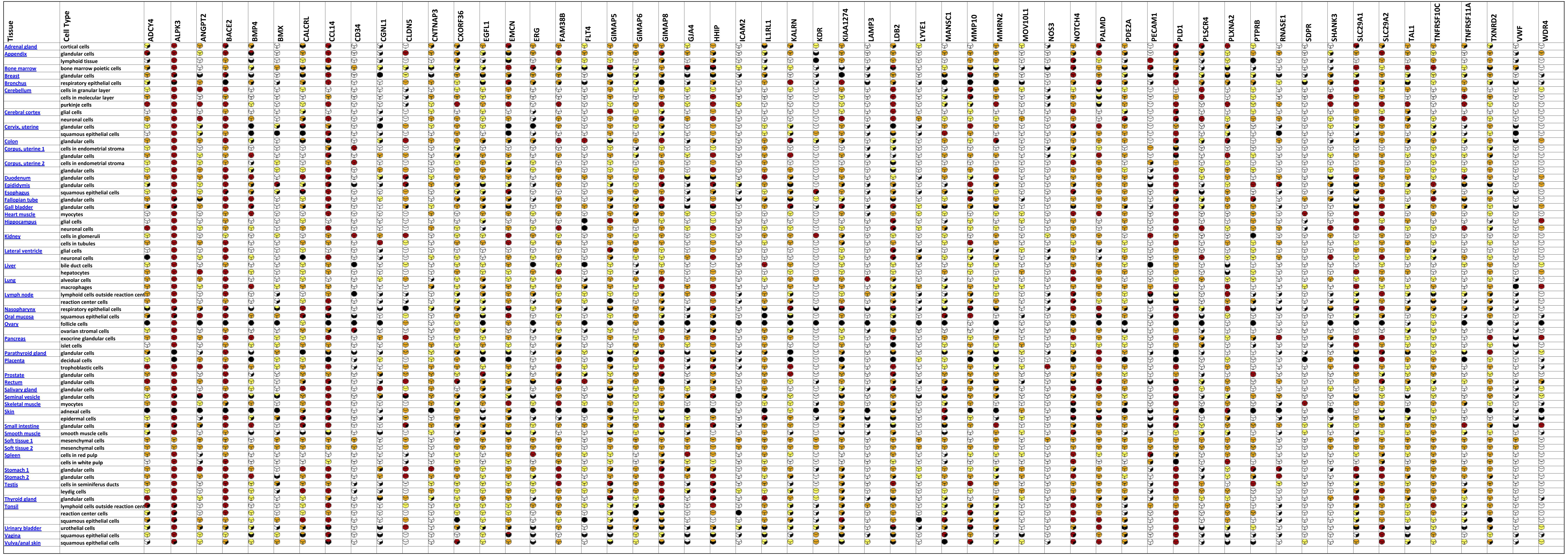
Tissue
Cell Type
ADCY4
ALPK3
ANGPT2
BACE2
BMP4
BMX
CALCRL
CCL14
CD34
CGNL1
CLDN5
CNTNAP3
CXORF36
EGFL1
EMCN
ERG
FAM38B
FLT4
GIMAP5
GIMAP6
GIMAP8
GJA4
HHIP
ICAM2
IL1RL1
KALRN
KDR
KIAA1274
LAMP3
LDB2
LYVE1
MANSC1
MMP10
MMRN2
MOV10L1
NOS3
NOTCH4
PALMD
PDE2A
PECAM1
PLD1
PLSCR4
PLXNA2
PTPRB
RNASE1
SDPR
SHANK3
SLC29A1
SLC29A2
TAL1
TNFRSF10C
TNFRSF11A
TXNRD2
VWF
WDR4
Adrenal gland | cortical cells
Appendix | glandular cells
lymphoid tissue
Bone marrow | bone marrow poietic cells
Breast | glandular cells
Bronchus | respiratory epithelial cells
Cerebellum | cells in granular layer
cells in molecular layer
purkinje cells
Cerebral cortex | glial cells
neuronal cells
Cervix, uterine | glandular cells
squamous epithelial cells
Colon | glandular cells
Corpus, uterine 1 | cells in endometrial stroma
glandular cells
Corpus, uterine 2 | cells in endometrial stroma
glandular cells
Duodenum | glandular cells
Epididymis | glandular cells
Esophagus | squamous epithelial cells
Fallopian tube | glandular cells
Gall bladder | glandular cells
Heart muscle | myocytes
Hippocampus | glial cells
neuronal cells
Kidney | cells in glomeruli
cells in tubules
Lateral ventricle | glial cells
neuronal cells
Liver | bile duct cells
hepatocytes
Lung | alveolar cells
macrophages
Lymph node | lymphoid cells outside reaction cent
reaction center cells
Nasopharynx | respiratory epithelial cells
Oral mucosa | squamous epithelial cells
Ovary | follicle cells
ovarian stromal cells
Pancreas | exocrine glandular cells
islet cells
Parathyroid gland | glandular cells
Placenta | decidual cells
trophoblastic cells
Prostate | glandular cells
Rectum | glandular cells
Salivary gland | glandular cells
Seminal vesicle | glandular cells
Skeletal muscle | myocytes
Skin | adnexal cells
epidermal cells
Small intestine | glandular cells
Smooth muscle | smooth muscle cells
Soft tissue 1 | mesenchymal cells
Soft tissue 2 | mesenchymal cells
Spleen | cells in red pulp
cells in white pulp
Stomach 1 | glandular cells
Stomach 2 | glandular cells
Testis | cells in seminiferus ducts
leydig cells
Thyroid gland | glandular cells
Tonsil | lymphoid cells outside reaction cent
reaction center cells
squamous epithelial cells
Urinary bladder | urothelial cells
Vagina | squamous epithelial cells
Vulva/anal skin | squamous epithelial cells
